# Resolving the Early Divergence Pattern of Teleost Fish Using Genome-Scale Data

**DOI:** 10.1093/gbe/evab052

**Published:** 2021-03-19

**Authors:** Naoko Takezaki

**Affiliations:** Life Science Research Center, Kagawa University, Mikicho, Kitagun, Kagawa, Japan

**Keywords:** phylogeny, outgroup, teleost fish, sequence divergence, genome-scale data

## Abstract

Regarding the phylogenetic relationship of the three primary groups of teleost fishes, Osteoglossomorpha (bonytongues and others), Elopomorpha (eels and relatives), Clupeocephala (the remaining teleost fish), early morphological studies hypothesized the first divergence of Osteoglossomorpha, whereas the recent prevailing view is the first divergence of Elopomorpha. Molecular studies supported all the possible relationships of the three primary groups. This study analyzed genome-scale data from four previous studies: 1) 412 genes from 12 species, 2) 772 genes from 15 species, 3) 1,062 genes from 30 species, and 4) 491 UCE loci from 27 species. The effects of the species, loci, and models used on the constructed tree topologies were investigated. In the analyses of the data sets (1)–(3), although the first divergence of Clupeocephala that left the other two groups in a sister relationship was supported by concatenated sequences and gene trees of all the species and genes, the first divergence of Elopomorpha among the three groups was supported using species and/or genes with low divergence of sequence and amino-acid frequencies. This result corresponded to that of the UCE data set (4), whose sequence divergence was low, which supported the first divergence of Elopomorpha with high statistical significance. The increase in accuracy of the phylogenetic construction by using species and genes with low sequence divergence was predicted by a phylogenetic informativeness approach and confirmed by computer simulation. These results supported that Elopomorpha was the first basal group of teleost fish to have diverged, consistent with the prevailing view of recent morphological studies.

**Significance**

Molecular studies supported all the possible relationships of the three primary groups of teleost fish, Elopomorpha, Osteoglossomorpha, and Clupeocephala. Using four genome-scale data sets, this study showed that the constructed tree topologies were strongly affected by the species and genes used. By using species and genes that increase the accuracy of phylogeny construction by theoretical prediction and computer simulation, all the four genome-scale data sets supported the first divergence of Elopomorpha, leaving Osteoglossomopha and Clupeocephala in a sister relationship. This result indicates the importance of choice of appropriate species and genes to resolve the relationship at a particular node in phylogenomic studies.

## Introduction

**Fig. 1.**
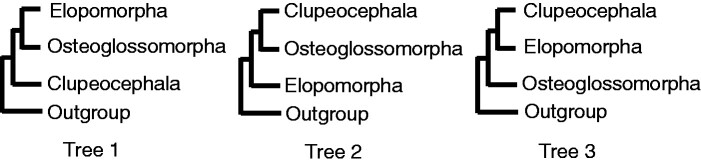
Three possible relationships of the three primary groups of teleost fish: Osteoglossomorpha, Elopomorpha, and Clupeocephala. Molecular studies supported Tree 1–3 are follows. Tree 1: Le et al. (1993), one nuclear gene (28S rRNA); Hurley et al. (2007), four nuclear protein-coding genes (*fzd8*, *hoxa11*, *sox11*, and *tyr*); Broughton (2010), mitochondrial genome; Chen et al. (2015), 4,682 nuclear protein-coding genes; Bian et al. (2016), 418 nuclear protein-coding genes; Hughes et al. (2018), 1,105 nuclear protein-coding genes (concatenated sequence); Vialle et al. (2018), 278 nuclear protein-coding genes. Tree 2: Normark et al. (1991), one mitochondrial protein-coding gene (*cytb*); Alfaro et al. (2009) and Santini et al. (2009), one nuclear protein-coding gene (*rag1*); Near et al. (2012), nine nuclear protein-coding genes (*Gly*t, *myh6*, *plagl2*, *Ptr*, *rag1*, *SH3PX3*, *sreb2*, *tbr1*, and *zic1*); Betancur-R et al. (2013), 20 nuclear protein-coding genes (*kiaa1239*, *ficd*, *myh6*, *panx2*, *plagl2*, *ptchd4* (=*ptr*), *ripk4*, *sidkey*, *snx33* (=*sh3px3*), *tbr1b* (=*tbr1*), and *zic1*, *hoxc6a* (intron), *svep1*, *vcpip*, *enc1*, *gtdc2* (=*glyt*), *gpr85* (=*sreb2*), *rag1*, *rag2*, and *rh*) and one mitochondrial gene (16S rRNA); Faircloth et al. (2013), 489 UCEs; Chen et al. (2014), three nuclear protein-coding genes (*rag1*, *EGR2B*, *EGR3*) and three mitochondrial genes (*COI*, 12S and 16S rRNA); Hughes et al. (2018), 1,105 nuclear protein-coding genes (gene-tree based approach). Tree 3: Forey et al. (1996), two mitochondrial genes (12S and 16S rRNA) and one nuclear gene (18S rRNA); Inoue et al. (2001, 2003, 2004), mitochondrial genome; Obermiller and Pfeifer (2003), two mitochondrial genes (12S and 16S rRNA); Johnson et al. (2012) and Lavoué et al. (2012), mitochondrial genome; Austin et al. (2015), 177 nuclear protein-coding genes.

With the advent of phylogenomic methods the resolution of ray-finned fish phylogeny has progressed in recent years. Early branching patterns of major clades of extant ray-finned fishes (Actinoptergii) (polypterids [e.g., bichir], chondrosteans [e.g., sturgeon and paddlefish], holosteans [lepisosterids {e.g., gar} and amiids {e.g., bowfin}], and teleosts [e.g., herring and salmon]) have been resolved molecularly as well as morphologically (Near et al. 2012; Betancur-R et al. 2013, 2017; Hughes et al. 2018). It is now broadly agreed that extant teleost fishes consist of three primary groups: Osteoglossomorpha (bonytongues; arawana and their relatives), Elopomorpha (eels, tarpons, and their relatives), and Clupeocephala (the remaining teleosts) (e.g., Nelson et al. 2016; Betancur-R et al. 2017; Hilton and Lavoué 2018). Regarding the phylogenetic relationships of these groups, morphological studies supported two hypotheses (Trees 2 and 3 in fig. 1). Early studies hypothesized the first split of Osteoglossomorpha from the other two (Tree 3, Patterson 1997; Patterson and Rosen 1997). The recent prevailing view is the first split of Elopomorpha (Tree 2, Arratia 1991; Li and Wilson 1996; Shen 1996; Arratia 1997; Zhang 1998; Diogo 2008; Arratia 1999, 2000, 2010) and its earliest diversification in the Late Jurassic (Arratia 1997, 2000, 2010) (see Wiley and Johnson 2010; Hilton and Lavoué 2018).

Molecular studies have supported all the possible relationships of the three groups (Trees 1–3, fig. 1). Early studies from the 1990s to early 2010s used a small number of mitochondrial and/or nuclear genes or mitochondrial genome data (Tree 1, Le et al. 1993; Hurley et al. 2007; Broughton 2010; Tree 2, Normark et al. 1991; Alfaro et al. 2009; Santini et al. 2009; Near et al. 2012; Betancur-R et al. 2013; Chen et al. 2014; Tree 3, Forey et al. 1996; Inoue et al. 2001, 2003; Obermiller and Pfeifer 2003; Inoue et al. 2004; Johnson et al. 2012; Lavoué et al. 2012). However, even in recent studies from the mid-2010s that used genome-scale data of more than a hundred genes or genomic regions supported all the possible relationships for the three groups (Tree 1, Chen et al. 2015; Bian et al. 2016; Hughes et al. 2018 [concatenated sequence]; Vialle et al. 2018; Tree 2, Faircloth et al. 2013; Hughes et al. 2018 [gene-tree based approach]; Tree 3, Austin et al. 2015; see details in the legend of fig. 1). The reason why the results varied among the studies is not known.

It is known that constructed tree topologies are affected by the species and genes (or genomic regions) included (e.g., rich vs. poor taxon sampling, fast- vs. slowly evolving species and genes, high vs. low composition bias), and the methods used (models of sequence evolution and partition schemes) (Stefanović et al. 2004; Philippe, Delsuc, et al. 2005; Shen et al. 2016). However, studies with genome-scale data have frequently supported contradictory results with high statistical support (Doyle et al. 2015), for example, for yeast (Rokas et al. 2003; Phillips et al. 2004), insects (von Reumont et al. 2012; Dell’Ampio et al. 2014), and metazoan lineages (Philippe, Lartillot, et al. 2005; Dunn et al. 2008; Philippe et al. 2009; Nosenko et al. 2013; Pisani et al. 2015; Whelan et al. 2015), as well as the relationships of the primary groups of teleost fishes. Therefore, in order to resolve the phylogenetic relationship it is important not only to use large amount of data, but also to use appropriate data of species and genes or genomic regions as well as methods (e.g., Kopfstein et al. 2017; Dornburg et al. 2019).

This study analyzed genome-scale data of protein-coding genes from three recent studies (Chen et al. 2015, 4,682 genes for 15 species of teleost fish and outgroups; Bian et al. 2016, 418 genes for 12 species; Hughes et al. 2018, 1,105 genes for 303 species), and ultraconserved elements (UCEs) (Faircloth et al. 2013, 489 UCEs for 27 species) (table 1 and supplementary table S1, Supplementary Material online), and investigated the effects of using different outgroups, species included in the taxonomic groups, different genes or genomic regions, and the substitution models on tree topology and why the supported relationships among the three primary groups of teleost fishes were different in these studies. The resolving power of the branching pattern among the three groups for the data was examined by computer simulation and the phylogenetic informativeness (PI) approach which provides theoretical prediction of the resolution power with respect to the extent of sequence divergence (Townsend 2007; López-Giráldez and Townsend 2011).

**Table 1:** Data Analyzed in this Study

Data	Bian	Chen				Hughes	Faircloth
Subset		Total	Teleost	Slow1000	Slow500		
No. species							
Total	12	15	15	15	15	30	27
(per locus)	—	—	—	—	—	25.5 ± 4.0	22.4 ± 3.0
Outgroup	2	4	4	4	4	12	4
Elopomorpha	2	1	1	1	1	4	2
Osteoglossomorpha	3	1	1	1	1	6	2
Clupeocephala	5	9	9	9	9	8	19
No. loci							
This study	412	772	542	190	96	1,062	278
Original study	418	4,682	3,834	1,000	500	1,105	491
No. sites (total)	166,669	318,449	240,681	100,624	56,974	166,583	149,246
No. parsimony informative sites	38,555	86,553	70,261	13,319	5,712	74,816	29,551

Note.—Data source: Bian, Bian et al. (2016); Chen, Chen et al. (2015); Hughes, Hughes et al. (2018); Faircloth, Faircloth et al. (2013).

## Materials and Methods

### Sequence Data Used

Amino-acid sequence data from three previous studies and nucleotide sequence from one study were analyzed (table 1 and supplementary table S1, Supplementary Material online). The data from Bian et al. (2016) were provided by the authors. Out of 418 genes for 12 species (coelacanth [*Latimeria chalumnae*]) and eight ray-finned fish, including one nonteleost fish (gar [*Leipdosteus oculatus*]) and seven teleost fishes [three Osteoglossomorpha (arawana or Asian bonytongue [*Scleopages formosus*], butterflyfish [*Pantodon buchholzi*], and knifefish [*Papyrocranus afer*]), two Elopomorpha (European eel [*Anguilla anguilla*], tarpon [*Megalops cyprinoides*]), five Clupeocephala (zebrafish [*Danio rerio*], electric eel [*Electrophorus electricus*], medaka [*Oryzias latipes*], fugu [*Takifugu rubripes*], and stickleback [*Gasterosteus aculeatus*]). Six genes whose number of shared amino-acid sites <50 were excluded. Thus, a set of 412 genes from the 12 species was used for the analyses (table 1 and supplementary table S15, Supplementary Material online).

Data from Chen et al. (2015), Hughes et al. (2018), and Faircloth et al. (2013) were downloaded from the Dryad Digital Repository. In the data from Chen et al. (2015), there were amino-acid sequences of 14 ray-finned fish: 11 teleost fish, including one Elopomorpha (Japanese eel [*Anguilla japonica*]), one Osteoglossomorpha (silver arawana [*Osteoglossum bicirrhosum*]), nine Clupeocephala species (zebrafish [*D. rerio*], catfish [*Ictalurus punctatus*], tetra [*Astyanax mexicanus*], cod [*Gadus morhua*], tilapia [*Oreochromis niloticus*], platyfish [*Xiphophorus maculatus*], medaka [*O. latipes*], stickleback [*G. aculeatus*], fugu [*T. rubripes*]), and three nonteleost fish (gar [*L. oculatus*], sturgeon [*Acipenser transmontanus*], and bichir [*Polypterus senegalus*]) (supplementary table S15, Supplementary Material online). The genes that included all 14 ray-finned fish species and the coelacanth (*L. chalumnae*) were extracted from the total gene set (4,682 genes) and those with <50 shared amino-acid sites were excluded (Total set, 772 genes). Within the Total set, genes included in the data set in which teleost species formed a monophyletic cluster, the top-1000 and -500 slowly evolving gene sets (Chen et al. 2015) were extracted: Teleost set (542 genes), Slow1000 set (190 genes), and Slow500 set (96 genes). The sets of top-200 and -100 slowly evolving genes were created by choosing the genes with short total branch lengths (TBLs) estimated for the trees of 15 species (data not shown). However, the results were essentially the same as those of the Slow1000 and Slow500 sets. Therefore, it was decided to use the Slow1000 and Slow500 sets.

In the Hughes et al. (2018) data, there were 1,105 individual genes. The individual genes contained 305 species in total: Frog (*Xenopus tropicalis*), coelacanth (*L. chalumnae*), lungfish (*Protopterus aethiopicus*), 10 nonteleost ray-finned fishes (three Polypteriformes, four Acipenseriformes, four Holostei [one Amiiformes, three Lepisosteiformes]), and 292 teleost fishes (seven Elopomorpha, six Osteoglossomorpha, and 279 Clupeocephala species) (supplementary tables S1, S16, and S17, Supplementary Material online). Out of 1,105 genes, six genes that contained no Osteoglossomorpha sequences were excluded (1,099-gene set) (supplementary tables S16 and S17, Supplementary Material online). Because the focus of this study is to resolve the relationships of Elopomorpha, Osteoglossomorpha and Clupeocephala, nine Clupeocephala species (Atlantic herring [*Clupea harengus*], golden-line barbel [*Sinocyclocheilus grahami*], red-bellied piranha [*Pygocentrus nattereri*], northern pike [*Esox lucius*], grayling [*Thymallus thymallus*], silver eye [*Polymixia japonica*], blackbar soldierfish [*Myripristis jacobus*], yellowfin tuna [*Thunnus albacares*], and northern snakehead [*Channa argus*]) that have low proportion of missing data and relatively low divergence were selected. Three Elopomorpha species (*Gymnothorax reevesii*, *Conger cinereus*, *Kaupichthys hyoproroides*), and one outgroup (*Acipenser naccarii*) which appeared in a small number of loci (*≤*171) were excluded (30 species in total). From the 1,099-gene set, loci in which some species have unusually long branch from the common ancestral node of teleost fish (>3 substitutions per site) and whose number of sites was <50 were excluded (1,062 loci) (table 1 and supplementary table S1, Supplementary Material online) (Hughes data).

Although nucleotide sequence data were available for the Bian data and Hughes data, this study analyzed amino-acid sequence data, because synonymous nucleotide sites were likely to be subjected to saturation due to the long time after separation of Elopomorpha, Osteoglossomorpha, and Clupeocephala (>250 My, e.g., Near et al. 2012; Hughes et al. 2018) and the amino-acid sequence whose number of states is 20 is less likely to produce noise than nucleotide sequence whose number of states is four. Multiple substitutions which are not correctly identified can generate spurious phylogenetic signals (e.g., Philippe, Delsuc, et al. 2005; Philippe et al. 2011). Indeed, estimated branch lengths for the third codon positions where most of the substitutions are synonymous were 5–7 times longer than those for the first and second codon positions where most of the substitutions are nonsynonymous (supplementary table S18, Supplementary Material online). The peaks of PI profile which indicates the resolving power of the branching pattern (see below) for the third codon positions were located at the shallow range of the teleost phylogeny (supplementary fig. S13, Supplementary Material online). The test of substitution saturation for nucleotides (Steel et al. 1993) by DAMBE7 (Xia 2018) indicated the sign of saturation for the third codon positions, whereas there was no sign of saturation for the first and second codon positions (supplementary table S18, Supplementary Material online).

Estimated branch lengths for amino-acid sequences were ∼70% longer than those for the first and second codon positions (supplementary table S18, Supplementary Material online) and the peak of the PI profile was at the shallower region of the teleost fish phylogeny (supplementary fig. S13, Supplementary Material online). However, the signal and noise analysis (Townsend et al. 2012) that takes into accounts the number of states of the sequence data for the tree of the four taxa, Osteoglossomorpha, Elopomorpha, Clupeocephala, and outgroup, predicted that the probability of obtaining noise for resolution of the branching pattern is higher for the first and second codon positions than amino-acid data, though the probability of obtaining signal for resolution was the same for both kinds of the data (supplementary table S18, Supplementary Material online).

In UCE data from Faircloth et al. (2013), there were four outgroups (bichir, lake sturgeon [*Acipenser fluvescens*], bowfin [*Amia calva*], and gar), two Elopomorpha (*Megalops* sp. and slender giant moray [*Strophidon sathete*]) and two Osteoglossomorpha (silver arawana and butterflyfish) and 19 Clupeocephala species (supplementary table S15, Supplementary Material online). Of the 491 UCE loci in the downloaded data, 278 loci that contained at least one species in each of the four groups (outgroup, Elopomorpha, Osteoglossomorpha, and Clupeocephala) (supplementary table S1, Supplementary Material online) were used for gene-tree based approach.

### Phylogenetic Analyses

Phylogenetic trees were constructed using the maximum likelihood (ML) method with RAxML 8.2.12 (Stamatakis 2014). The fit of substitution models was examined for each gene data set of the Bian, Chen, and Hughes data, fixing the tree topologies to the those constructed for the concatenated sequences using four substitution models, JTT (Jones et al. 1992), LG (Le and Gascuel 2008), Dayhoff (Dayhoff et al. 1978), WAG (Whelan and Goldman 2001), MTMAM (Yang et al. 1998), with or without the use of empirical amino-acid frequencies (+F), assuming that the rate was gamma-distributed across sites with four discrete categories (+G). The fit was tested by AIC (Akaike information criterion), which is defined as –2 *L *+* *2*k*, where *L* is a log-likelihood value and *k* is the number of parameters. The majority of genes showed the best fit to JTTG or JTTFG for all data sets (supplementary table S19, Supplementary Material online). Therefore, JTTFG and JTTG were used for the construction of phylogenetic trees with concatenated sequences and individual genes, respectively, GTRG (general time-reversible model +G) was also used for the construction of phylogenetic trees with concatenated sequences. For the Hughes data likelihood values were only computed for three possible tree topologies corresponding to Tree 1–3, by fixing the branching patterns of the remaining parts to those constructed using JTTFG. The tree topology with the highest likelihood was considered the best tree. AU test was conducted using CONSEL (Shimodaira and Hasegawa 2001). Using GTRG model, the bootstrap tests were not completed because they took a prohibitive amount of time with this data. Bayesian analysis by the CAT model (Lartillot and Philippe 2004) could not be used because of the limitation of computation time. For the UCE data (Faircloth et al. 2013) GTRG model was used.

Using the trees constructed for individual loci (gene trees), species phylogeny was estimated by ASTRAL 5.6.3 (Zhang et al. 2018). A total of 500 bootstrap replications was conducted when 500 or more loci were in the set. When there were <500 loci, the bootstrap replications were conducted for the number of loci in the set.

The optimal partition scheme was searched for the Bian and Chen data using PartitionFinder 1.1 (Lanfear et al. 2012) with the AICc (corrected for small sample size) and Bayesian Information Criterion (BIC) criterion. AICc = 2 *L *+* *2*k*(*k *+* *1)/(*n*–*k*–1) and BIC = –2 *L* + *k*ln (*n*), where *n* is the number of sites. To reduce the computation time, the genes were divided into three groups: Those that had the best fit with 1) JTTG and JTTFG, 2) LGG and LGFG, and 3) other substitution models examined. For the first two groups, the search algorithm “rcluster” was used, restricting the substitution models to 1) JTTG and JTTFG and 2) LGG and LGFG. For the third group, the search algorithm “greedy” was used with all the substitution models available (“all_protein” option). The partitioned analysis was not applied to the Hughes data because of the large numbers of loci (1,062) and species (30).

### Computer Simulation

Amino-acid sequence data for 300 sites were generated by assuming that the model trees corresponding to Trees 1–3 fixing the remaining branching patterns as those estimated for the concatenated sequence for the Bian, Chen, and Hughes data (supplementary figs. S5 and S6, Supplementary Material online), and assuming JTTFG with Seq-Gen version 1.3.4 (Rambaut and Grass 1997). After the 12 sequences for the Bian data, 15 sequences for the Chen data, and 30 sequences for the Hughes data were generated, different combinations of sequences of outgroups, Osteoglossomorpha, Elopomorpha, and Clupeocephala species were extracted. The likelihoods for the tree topologies corresponding to Trees 1–3 for the extracted sequences were computed with JTTFG. The tree topology with the highest likelihood value was considered the best tree. A total of 1,000 replications was conducted in each case. In the preliminary study, computer simulations were conducted by assigning observed amino-acid frequencies of species using INDELible (Fletcher and Yang 2009). However, the results were essentially the same as those conducted by assuming JTTFG by Seq-Gen (data not shown).

### PI Approach

PI (Townsend 2007) was examined by profiles of PI along the sequence divergence obtained through PhyDesign (Mayrose et al. 2004; López-Giráldez and Townsend 2011). Site rates of substitution were computed by Hyphy 2.5.26 (Kosakovsky Pond et al. 2005), assuming JTTG for amino-acid sequences GTRG for nucleotide sequences. Branch lengths of the rooted tree (Tree 1) with the molecular clock were estimated by codeml in PAML 4.9j (Yang 2007) and the tree was regarded as the ultrametric tree. PI values were computed by multiplying site rates by the ratio of the TBLs of the ultrametric of the subsets of genes to that for the total gene set (Moeller and Townsend 2011; Dornburg, Townsend, Wang, et al. 2017) of the Chen and Hughes data.

## Results

### The Effect of Species Included in Phylogeny Construction

Many factors can distort constructed tree topologies: Use of a distantly related outgroup (e.g., Philippe, Delsuc, et al. 2005; Philippe et al. 2009; Takezaki and Nishihara 2016, 2017), the addition of distantly related taxa (deletion of fast-evolving taxa can increase the accuracy of phylogeny construction, e.g., Philippe, Delsuc, et al. 2005; Philippe, Lartillot, et al. 2005; Pisani et al. 2015), and compositional bias (Betancur-R et al. 2013; Cox et al. 2014; Li et al. 2014; Dornburg, Townsend, Brooks, et al. 2017). There was variation in the rates and amino-acid frequencies among species in the Bian, Chen, and Hughes data (supplementary tables S2 and S3, Supplementary Material online). The effect of species included in constructed tree topologies was investigated.

### Bian Data

**Fig. 2.**
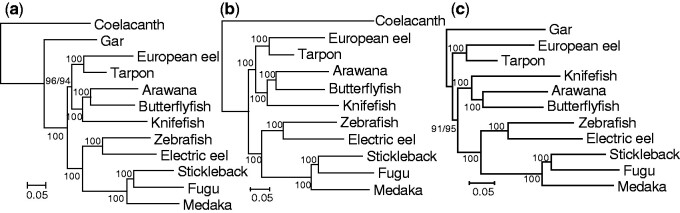
The phylogenetic trees constructed for concatenated sequences of the 412 gene set of the Bian data. JTTFG was used and 500 bootstrap replications were done. Coelacanth and gar were used as the outgroups in (*a*) coelacanth in (*b*), and gar in (*c*). The bootstrap probabilities (BPs) for GTRG are shown after the slash. In the cases where only one BP is shown, BPs for the two substitution models were the same.

In the Bian data, there were 12 species (two outgroups, three Osteoglossomorpha, two Elopomorpha, and five Clupeocephala). First, to determine the effect of the outgroup, phylogenetic trees were constructed with the concatenated sequences (fig. 2). By using both the coelacanth and gar as the outgroup, Tree 1 was constructed with a high bootstrap probability (BP) (96%) (fig. 2*a*), as in the results of Bian et al. (2016). Using only the distantly related coelacanth [branch length from the common ancestral node of teleost fish (*b_R_*) = ∼0.43] as the outgroup, Tree 1 was constructed with an even higher BP (100%) (fig. 2*b*). However, using only the closely related gar (*b_R_* = ∼0.21) as the outgroup, Tree 2 was constructed with a high BP as well (91%) (fig. 2*c*).

Next, phylogenetic trees were constructed by using different combinations of species in Osteoglossomorpha (arawana, butterflyfish, and knifefish) and Elopomorpha (European eel and tarpon) (supplementary table S4, Supplementary Material online). As in the case in which all species in Osteoglossomorpha and Elopomorpha were included (fig. 2), Tree 1 was constructed with both the coelacanth and gar or only the coelacanth as the outgroup, with a higher BP for the latter than the former. Tree 2 was constructed with only gar as the outgroup. However, there were the following tendencies: 1) by including arawana in Osteoglossomorpha, Tree 1 or Tree 3 was constructed even if only the gar was used as the outgroup (combinations 13–15, supplementary table S4, Supplementary Material online), 2) use of the tarpon rather than the European eel for Elopomorpha increased the BP supporting Tree 2 (e.g., combinations 8 and 9), and 3) with the use of knifefish for Osteoglossomorpha Tree 2 was constructed, even with both the coelacanth and gar as the outgroup (BP = 79–93%) (combinations 19–21). The BP supporting Tree 2 was higher (80–99%, combinations 7–11) than the cases where it was excluded (41–52%, combinations 5–6).

Consistent with the results above for different combinations of the five species in Clupeocephala (*b_R_* = 0.23–0.32) (supplementary table S5, Supplementary Material online), although Tree 1 was always constructed with the coelacanth or coelacanth and gar as the outgroup, Tree 2 was more often constructed by including species with shorter *b_R_* than species with longer *b_R_* using the gar as the outgroup (table 2).

**Table 2:** Summary of Tree Topologies Constructed for Different Combinations of Species of Clupeocephala in the Bian Data

Data	Subset	Outgroup	Best Tree					
			Tree 1		Tree 2		Tree 3	
			*N* ^a^	Branch Length^b^	*N* ^a^	Branch Length^b^	*N* ^a^	Branch Length^b^
Bian		Coelacanth, gar	30	0.278 ± 0.040	0		0	
		Coelacanth	30	0.278 ± 0.040	0		0	
		Gar	7	0.295 ± 0.297	23	0.274 ± 0.274	0	
Chen	Total	All	494	0.194 ± 0.009	0		14	0.197 ± 0.011
		Coelacanth	508	0.194 ± 0.009	0		0	
		Bichir	458	0.194 ± 0.009	0		50	0.190 ± 0.008
		Sturgeon	505	0.194 ± 0.009	0		3	0.189 ± 0.013
		Gar	33	0.192 ± 0.012	5	0.184 ± 0.013	470	0.194 ± 0.009
	Teleost	All	503	0.211 ± 0.01	0		5	0.206 ± 0.014
		Coelacanth	508	0.211 ± 0.01	0		0	
		Bichir	425	0.211 ± 0.009	0		83	0.208 ± 0.007
		Sturgeon	505	0.211 ± 0.009	0		3	0.205 ± 0.015
		Gar	287	0.209 ± 0.009	4	0.201 ± 0.014	217	0.213 ± 0.009
	Slow1000	All	0		0		508	0.089 ± 0.004
		Coelacanth	261	0.089 ± 0.005	1	0.097	246	0.090 ± 0.004
		Bichir	59	0.090 ± 0.006	1	0.079	448	0.089 ± 0.004
		Sturgeon	327	0.090 ± 0.004	159	0.088 ± 0.004	22	0.090 ± 0.003
		Gar	0		1	0.079	507	0.089 ± 0.004
	Slow500	All	387	0.063 ± 0.003	1	0.057	120	0.065 ± 0.003
		Coelacanth	480	0.064 ± 0.003	1	0.068	27	0.063 ± 0.004
		Bichir	391	0.064 ± 0.004	1	0.057	116	0.064 ± 0.003
		Sturgeon	250	0.064 ± 0.003	258	0.063 ± 0.003	0	
		Gar	128	0.063 ± 0.003	54	0.061 ± 0.004	326	0.064 ± 0.003

Note.—All the species in Osteoglossomorpha and Elopomorpha were included.

a The number of different combinations of species in Clupeocephala.

b Average branch length from the common ancestral node of teleost fish.

The branch length from the common ancestral node of teleost fish (*b_R_*) to the coelacanth (∼0.43) was approximately two times as long as that to the gar (∼0.21). In Elopomorpha, the *b_R_* of the European eel (∼0.18) was much longer than that of the tarpon (∼0.10) (supplementary table S2, Supplementary Material online). Although the *b_R_* of arawana (∼0.19) was similar to that for the other species in Osteoglossomorpha (0.17–0.22), it had divergent amino-acid frequencies (supplementary table S3, Supplementary Material online). Therefore, there were tendencies for Tree 2 to be constructed or supported strongly by using species with shorter *b_R_* and less divergent amino-acid frequencies, whereas Tree 1 was constructed or supported strongly by using species with longer *b_R_* and divergent amino-acid frequencies, though it is not clear why Tree 2 was constructed or supported more strongly by the use of knifefish.

The results of the analyses of the Bian data suggested that Tree 2 likely reflects the actual branching pattern of the three primary groups of teleost fish because 1) computer simulation, discussed below, will show that the probability of obtaining correct tree topologies became higher by including species with shorter *b_R_* than species with longer *b_R_* and 2) the heterogeneity of amino-acid frequencies among species was an important factor that distorted tree topologies (Shen et al. 2016; Dornburg et al. 2019). Although Tree 3 was constructed in cases where some species in Osteoglossomorpha and Elopomorpha were excluded, it will be shown below in the computer simulation that the probability of obtaining the correct tree topologies (*N_C_*: the number of replications in which the correct tree topology was obtained) decreased by including a smaller number of species (supplementary fig. S1, Supplementary Material online).

### Chen Data

**Fig. 3.**
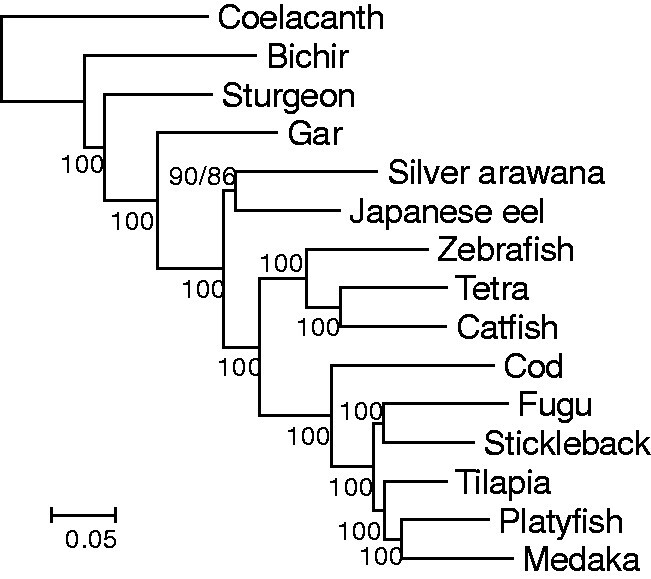
The phylogenetic trees constructed for concatenated sequences of the Total set from the Chen data. JTTFG was used and 500 bootstrap replications were done. Coelacanth, bichir, sturgeon, and gar were used as the outgroup. The BP for GTRG is after the slash if it is different from that for JTTFG.

In the Chen data, there were four outgroups, nine species in Clupeocephala and one species in Osteoglossomorpha and Elopomorpha (fig. 3). Phylogenetic trees were constructed with concatenated sequences of the four sets (Total, Teleost, Slow1000, Slow500). Relative *b_R_* values of species were similar among the four sets, whereas the TBLs were slightly longer (∼7%) for the Teleost set than for the Total set and much shorter for the Slow1000 (47%) and Slow500 (34%) sets (supplementary table S2, Supplementary Material online).

First, phylogenetic trees were constructed by using different outgroups (table 3 and supplementary fig. S2, Supplementary Material online). Tree 1 was generally constructed with all four outgroup species, and relatively distantly related outgroup species, that is, coelacanth, bichir, and sturgeon, were considered separately. Similar to the results of the Bian data, the BPs supporting Tree 1 were the highest with coelacanth as the outgroup and they tended to be lower with the other outgroups. Tree 3 was constructed with the most closely related outgroup, gar (*b_R_* = 0.14), which was similar in some cases of the Bian data, in which some of the species were excluded in Osteoglossomorpha and Elopomorpha (supplementary table S5, Supplementary Material online). Note that Tree 3 was also constructed with all outgroup species, coelacanth, or bichir as the outgroup for the Slow1000 set. Tree 2 was constructed in one case with sturgeon as the outgroup for the Slow500 set (table 3). BPs supporting the tree topologies constructed for the Slow 500 set were generally low (≤71%), which was probably because of the small number of genes in this set (table 1) and the low sequence divergence (supplementary table S2, Supplementary Material online).

**Table 3:** Summary of Tree Topologies Constructed for Concatenated Sequences of the Chen Data with Different Outgroups

Data Set	Outgroup									
	All		Coelacanth		Bichir		Sturgeon		Gar	
	Tree	BP	Tree	BP	Tree	BP	Tree	BP	Tree	BP
Total	1	90	1	100	1	61	1	99	3	88
Teleost	1	94	1	100	3	52	1	96	3	53
Slow1000	3	90	3	65	3	96	1	45	3	97
Slow500	1	57	1	71	1	46	2	68	3	47

Note.—BP, bootstrap probability. The values are shown in percentage. JTTFG was used.

Next, phylogenetic trees were constructed with different combinations of nine species in Clupeocephala. The constructed tree topologies were mostly Tree 1 or Tree 3 for the Total and Teleost sets. However, for the Slow1000 and Slow500 sets, Tree 2 was constructed for several combinations with sturgeon as the outgroup for the Slow1000 set (250) and sturgeon (258) or gar (54) as the outgroup for the Slow500 set, whereas for those with all or distantly related outgroups (coelacanth and bichir), the tree topologies were mostly Tree 1 or Tree 3 (table 2 and supplementary table S6, Supplementary Material online). In the cases in which Tree 2 was constructed, *b_R_* of species included in Clupeocephala was on average shorter than those cases in which Tree 1 or Tree 3 was constructed, as with the Bian data (table 2).

The power to resolve the phylogenetic relationships for the four sets with respect to sequence divergence was examined by the PI approach (Townsend 2007). The peaks of the power of the Total and Teleost sets were in the shallow range in the teleost phylogeny, whereas those of the Slow1000 and Slow500 sets were in relatively deep ranges (supplementary fig. S3, Supplementary Material online). Therefore, this approach suggested that in terms of sequence divergence, the Slow1000 and Slow500 sets had greater power to resolve the basal relationships of teleost fish than did the Total and Teleost sets.

For different combinations of species in Clupeocephala for the Slow1000 and Slow500 sets, Tree 2 was constructed more often with sturgeon than with gar as the outgroup. Although the *b_R_* of sturgeon was a little longer (∼20%) than that of gar (supplementary table S2, Supplementary Material online), amino-acid frequencies of gar were more divergent than those of the sturgeon (supplementary table S3, Supplementary Material online). Therefore, consistent with the Bian data, Tree 2 tended to be constructed by including species with a shorter *b_R_* and fewer divergent amino-acid frequencies in the Chen data.

### Hughes Data

**Fig. 4.**
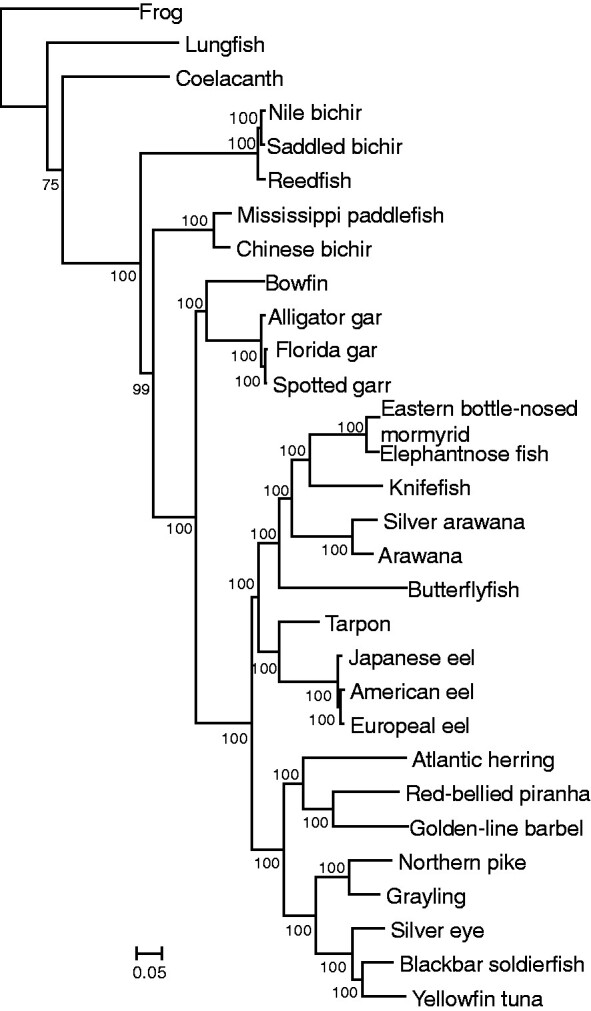
The phylogenetic tree constructed for concatenated sequence of the Hughes data. All the outgroup species were included. JTTFG was used and 500 bootstrap replications were done.

In the Hughes data, there were 30 species in total (12 outgroups, six Osteoglossomorpha, four Elopomorpha, and nine Clupeocephala), though the number of species was variable per locus (25.5 ± 4.0) (supplementary table S1, Supplementary Material online). Phylogenetic trees of concatenated sequences were constructed with all outgroups, frog, lungfish, coelacanth, Polypteriformes (three species), Acipenseriformes (two species), and Holostei (four species), as the outgroup separately, the last three corresponding to bichir, sturgeon, and gar in the Chen or Bian data, respectively (table 4). Although sequence divergence (*b_R_*) of nine Clupeocephala species of the Hughes data was similar to the Bian data and 1.5 times larger than the Total set of the Chen data, sequence divergence of outgroups, Elopomorpha, and Osteoglossomorpha in the Hughes data was 1.5 times and >2 times greater than that of the Bian data and the Total set of the Chen data, respectively. The *b_R_*s of frog and lungfish were 30% and 7% greater than that of the coelacanth, but the *b_R_* values of the other three outgroups relative to that of the coelacanth largely corresponded to those in the Bian and Chen data (supplementary table S2, Supplementary Material online). Similar to the results using the Bian and Chen data, Tree 1 was constructed using all the outgroups (fig. 4) or relatively distant outgroups with high statistical support (BP = 100%). When the most closely related Holostei was used as the outgroup, Tree 1 was also constructed, but the statistical support was not high (BP = 73%) (table 4).

**Table 4:** The Trees Constructed for Concatenated Sequences of the Hughes Data Using Different Outgroups

Outgroup	Tree	BP
All	1	100
Frog	1	100
Lungfish	1	100
Coelacanth	1	100
Polypteriformes	1	100
Acipenseriformes	1	100
Holostei	1	73

Note.—BP, bootstrap probability.

In Osteoglossomorpha, butterflyfish had divergent amino-acid frequencies and a higher *b_R_* (25%) than the other species. In Elopomorpha which consisted of three eel species and tarpon, the *b_R_*s of the eels were ∼40% longer than that of the tarpon (supplementary tables S2 and S3, Supplementary Material online). By excluding the butterflyfish, eels, and tarpon, phylogenetic trees were constructed using different outgroups (supplementary table S7, Supplementary Material online). When the butterflyfish or tarpon was excluded, tree topologies and the statistical support remained essentially the same. However, by excluding the eels, Tree 2 was constructed with high statistical support (BP = 97%) using Holostei as the outgroup. Tree 1 was constructed using the other distant outgroups with high statistical support (BP ≥ 97%), but including all the outgroups, the statistical support was low (BP = 71%).

**Fig. 5.**
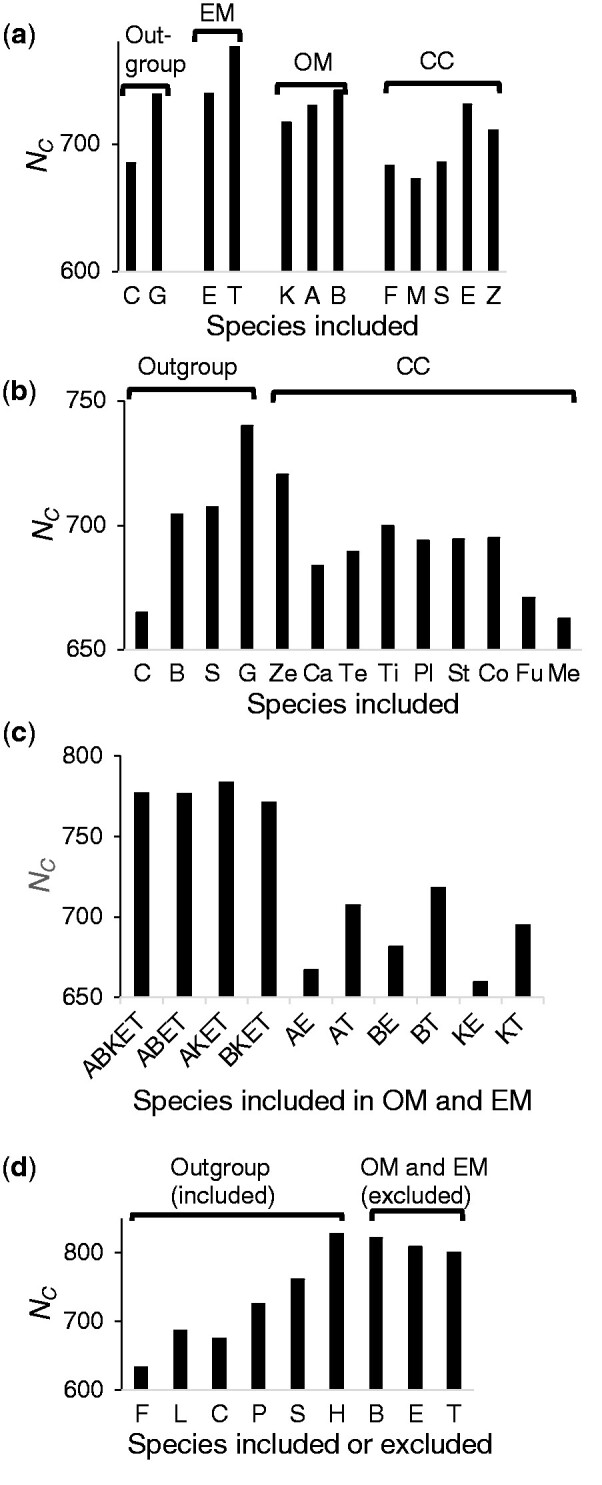
The number of replications in which correct tree topologies are obtained (*N_C_*) in computer simulation. OM, Osteoglossomorpha; EM, Elopomorpha; CC, Clupeocephala. (*a*) For the Bian data. Species in the four groups, outgroup, Elopomorpha, Osteoglossomorpha, and Clupeocephala, were changed. Outgroup: C, coelacanth, G, gar. Osteoglossomorpha: K, Knifefish, A, arawana, B: butterflyfish. Elopomorpha: E, European eel, T, tarpon. Clupeocephala: Z, zebrafish, E, Electric eel, S, stickleback, F, fugu, M, medaka. (*b*) For the Total set of the Chen data. Species in outgroup and Clupeocephala were changed. Outgroup: C, coelacanth, B, bichir, S, sturgeon, and G, gar. Clupeocephala: Ze, zebrafish, Ca, catfish, Te, tetra, Ti, tilapia, St, stickleback, Pl, platyfish, Co, cod, Fu, fugu, and Me, medaka. When species of one group was changed, all the species in the other groups were included. (*c*) For the Bian data. The *N_C_*s for the cases in which multiple species were included in Osteoglossomorpha, and Elopomorpha and those for the cases only one species was included in the two groups. All the outgroup species and species in Clupeocephala were included. (*d*) For the Hughes data. When species in Osteoglossomorpha and Elopomorpha were changed, Holostei were used as the outgroup. F, frog; L, lungfish; C, coelacanth; P, Polypteriformes; S, Acipenseriformes; H, Holostei; B, butterflyfish; J, Japanese eel; E, European eel, American eel, and Japanese eel; T, tarpon. Tree 1 was used as the model tree. The results for all the three model trees are shown in supplementary figures S5 and S6, Supplementary Material online.

Correspondingly, the computer simulation below will show that the probability of obtaining the correct tree increased using closely related outgroups and including the less divergent tarpon than the eels when Holostei was used as the outgroup or all the outgroups were used. But the probability of obtaining the correct tree did not always increase when the other outgroups were used (fig. 5, supplementary fig. S4 and table S8, Supplementary Material online). Therefore, the results of the analysis of the Hughes data also suggested that Tree 2 reflects likely the actual branching pattern of the three primary groups of teleost fish.

### Partitioned Approach and Gene-Tree-Based Approach

In the above analyses, phylogenetic trees were constructed with concatenated sequences using the JTTFG model. Phylogenetic trees were constructed for concatenated sequences with a different substitution model (GTRG) and using the partitioned approach. For the Hughes data, only AU test was conducted for the GTRG model, by computing the likelihood values of the three tree topologies corresponding to Trees 1–3 because of the large numbers of loci and species. They were mostly the same as those of JTTFG, and BPs supporting the constructed tree topologies and *P* values of AU test were similar (supplementary table S7, Supplementary Material online).

In the gene-tree based approach by ASTRAL, tree topologies were the same as those for the concatenated sequences except one case in which Tree 3 was constructed with all the outgroups for the Hughes data instead of Tree 1 which was constructed for the concatenated sequence. Although the BPs in the gene-tree based approach were much lower (15.5–20% for the Bian data, 28.6–39.9% for the Chen data, and 6.7–16.8% for the Hughes data) (supplementary table S7, Supplementary Material online). This result suggested that the effects of incomplete lineage sorting, as well as the substitution models used and the partitioning on the constructed tree topologies, were small compared with that of species included and the sequence divergence.

### Computer Simulation

In the above analyses, the constructed tree topologies were affected by branch lengths, amino-acid frequencies of the included species, and the divergence of sequence data. The effects of branch lengths by the species included were examined by computer simulation generating sequence data for model trees corresponding to Trees 1–3 with branch lengths estimated from the Bian data, the four sets of the Chen data, and the Hughes data (supplementary figs. S5 and S6, Supplementary Material online) (see for all the results supplementary tables S8–S10, Supplementary Material online). In the following the results for which Tree 1 was used as the model tree (fig. 5) will be discussed. However, the results were similar among the cases in which Trees 1–3 were used as the model trees (supplementary figs. S4, S7, and S8, Supplementary Material online).

For the Bian data different species of the outgroup, Osteoglossomorpha, Elopomorpha, and Clupeocephala were included (fig. 5*a*). The numbers of replications in which the correct tree topologies were obtained (*N_C_*s) were higher when species with a shorter *b_R_*, such as gar as the outgroup or tarpon in Elopomorpha, was included than when species with a longer *b_R_*s, such as coelacanth or European eel, were included. Although the differences in the *b_R_*s of species in Osteoglossomorpha and Clupeocephala were relatively small, there was a tendency for the *N_C_* to become higher when including species with a shorter *b_R_* (fig. 5*a* and supplementary fig. S7, Supplementary Material online). There was a positive correlation between the difference in *N_C_*s (*ΔN_C_*) and that of the *b_R_*s to species included (*Δb_R_*) (supplementary fig. S9*a* and table S11, Supplementary Material online).

For the four sets of the Chen data, the outgroups and species in Clupeocephala were changed. Even though the extent of the sequence divergence of the four sets varied, the *N_C_*s tended to be higher when including species with a shorter *b_R_* in the result of the four sets (supplementary fig. S9*b*–*e* and table S11, Supplementary Material online), similar to the Bian data.

Although the Bian data included multiple species in Osteoglossomorpha and Elopomorpha, only one species was available in these groups in the Chen data. *N_C_*s in the cases in which multiple species were included in Osteoglossomorpha and Elopomorpha were much higher than those in which one species was included for these groups in the Bian data (fig. 5*c* and supplementary fig. S1, Supplementary Material online). This result was consistent with the theoretical study (Townsend and López-Giráldez 2010) and computer simulation (e.g., Hillis 1998; Heath et al. 2008).

For the Hughes data, the outgroups were changed and the butterflyfish, eels, and tarpon which had different *b_R_*s in Osteoglossomopha and Elopomorpha were excluded. As the results of the Bian and Chen data, the *N_C_* tended to become higher when the outgroups with shorter *b_R_s* were used. In contrast, when the tarpon with a shorter *b_R_* than the eels was included (by excluding the eels), the *N_C_*s were sometimes lower than those when the eels were included (supplementary table S8, Supplementary Material online). However, with all the outgroups or Holostei as the outgroups, the *N_C_*s were always higher when the tarpon was included than when the eels were included (fig. 5*d*). When the butterflyfish, which had a longer *b_R_* than the other Osteoglossomorpha species, was excluded, the *N_C_*s slightly decreased in general, but remained similar. This may be because there were six species of Osteoglossomorpha in the Hughes data and exclusion of one species had a small effect on the tree topology.

### UCE Data

Although concatenated sequences of protein-coding genes in the three previous studies (Chen et al. 2015; Bian et al. 2016; Hughes et al. 2018) all supported Tree 1, that of UCE data supported Tree 2 with high statistical support (Faircloth et al. 2013; BP = 100% in supplementary fig. S10, Supplementary Material online). One of the reasons for this difference in constructed tree topologies appears to be the relatively low sequence divergence of the UCE data. The *b_R_*s of UCE data were on average ∼50% of those of the Bian data, 30–50% of the Hughes data, and 95% and 90% of the Total and Teleost sets of the Chen data (supplementary table S2, Supplementary Material online). Although the *b_R_*s of the UCE data were approximately two to three times longer than those of the Slow1000 and Slow500 sets of the Chen data, the UCE data contained two species in both Elopomorpha and Osteoglossomorpha, in contrast to the Chen data that included only one species in these groups.

To see the effect of the species included on the UCE data, phylogenetic trees were constructed for different combinations of species in Elopomorpha and Osteoglossomorpha, as well as the outgroups (supplementary table S7, Supplementary Material online). For most of the combinations, Tree 2 was constructed. However, when only one species was included in Elopomorpha (slender giant moray) and Osteoglossomorpha (silver arawana) for Osteoglossomorpha, Tree 3 was constructed in some cases, although BPs supporting the tree topologies were low (52–64%). It should be noted that sequence divergence of the slender giant moray (*b_R_* = 0.11) was approximately two times higher than that of the other Elopomorpha species (*Megalops* sp.) (*b_R_* = 0.06), whereas the *b_R_* (= 0.11) of the silver arawana was shorter than that of the other Osteoglossomorpha species (butterflyfish, *b_R_* = 0.15) but the nucleotide frequencies of the former appeared more divergent than those of the latter (supplementary table S3, Supplementary Material online).

## Discussion

In this study, genome-scale sequence data of protein-coding genes from three previous studies and UCEs were analyzed, focusing on the relationships of the three primary groups of teleost fish: Osteoglossomorpha, Elopomorpha, and Clupeocephala. The resulting tree topologies were affected by species included in tree construction. By using species with a high divergence in sequences and amino-acid frequencies, Tree 1 in which Clupeocephala was the lineage that diverged first among the three groups was supported, as in the results of Bian et al. (2016), Chen et al. (2015), and in the tree constructed with the concatenated sequences in Hughes et al. (2018). However, by using species with low divergences in sequence data and amino-acid frequencies, Tree 2 in which Elopomorpha was the first group to have diverged tended to be consistently supported in all the data sets of these three studies, as in the result of Faircloth et al. (2013) in which sequence divergence of the UCE data set was much lower than those of the three studies. Computer simulation and the PI approach indicated that the accuracy of phylogeny construction increased with the use of species with lower divergence. This result suggested that Tree 2 reflects the actual branching pattern of the three primary groups of teleost fish, consistent with recent morphological studies (e.g., Arratia 2010).

### Divergence of Sequence of Gene Data

The PI approach (Townsend 2007) on the four sets of Chen data showed that in terms of sequence divergence, the Slow1000 or Slow500 sets had the peaks of power to resolve phylogenetic relationships nearer the basal node of teleost fish than did the Total and Teleost sets. However, the peaks of the Slow500 and Slow1000 sets still had shallower points than did the basal node of teleost fish (supplementary fig. S3, Supplementary Material online). Therefore, the effect of sequence divergence on the constructed tree topology was investigated by sorting loci by the TBL of the gene trees and creating a top-10, -50, and -100 gene set of low divergence for the Bian and Chen data. TBLs of these top gene sets were ∼10%, 20%, and 35% of concatenated sequences of the 412 gene set for the Bian data, and ∼20%, 50%, and 80% for the Chen data for the Slow500 set, respectively (table 5 and supplementary table S2, Supplementary Material online).

**Table 5:** The Phylogenetic Trees Constructed for Gene Sets with Small Sequence Divergence in the Bian and Chen Data

Data	Species Included			Total Branch Length						Amino-Acid Frequencies					
	Outgroup	Osteoglossomorpha	Elopomorpha	Top-10		Top-50		Top-100		Top-10		Top-50		Top-100	
		Tree	BP	Tree	BP	Tree	BP	Tree	BP	Tree	BP	Tree	BP
Bian	Coelacanth, gar	Butterflyfish, knifefish	European eel, Tarpon	2	35	2	75	3	60	—	—	2	92	2	64
	Coelacanth			—	—	3	70	1	65	—	—	3	52	1	76
	Gar			2	42	2	93	2	61	—	—	2	100	2	89
	Coelacanth, gar	Butterflyfish, knifefish	Tarpon	3	22	2	94	2	70	—	—	2	96	2	63
	Coelacanth			—	—	2	94	1	58	—	—	3	93	1	56
	Gar			2	52	2	98	2	96	—	—	2	99	2	83
	Coelacanth, gar	Arawana	European eel	3	44	3	89	3	75	3	74	3	85	3	69
	Coelacanth			2	56	3	70	1	66	—	—	3	78	1	57
	Gar			2	46	3	89	3	74	3	78	3	64	3	85
	Tree length*			0.220		0.489		0.766		0.265		0.549		0.866	
	Number of sites			2,674		18,585		41,384		3,001		17,716		37,007	
Chen	All	Silver arawana	Japanese eel	2	89	2	62	1	68	—	—	1	87	2	51
	Coelacanth			2	45	3	77	3	67	2	50	3	69	3	90
	Bichir			2	86	2	62	1	63	2	73	1	75	3	81
	Sturgeon			2	99	2	91	2	73	2	41	1	88	2	94
	Gar			2	68	2	78	2	45	2	57	1	45	2	61
	Tree length*			0.141		0.330		0.499		0.272		0.402		0.592	
	Number of sites			5,923		25,046		47,200		3,070		17,622		39,227	

Note.—Heterogeneity of amino acid frequencies was examined by average of pairwise *χ*^2^ value between species. Total branch length: Genes were sorted by total branch length of the tree. A hyphen “—” indicates that the constructed tree matched none of Tree 1–3. Tree length*: the proportion of the total branch length relative to the total gene set.

Phylogenetic trees were constructed for concatenated sequences of these gene sets by changing species included as the outgroup and for the Osteoglossomorpha and Elopomorpha (table 5). Tree 2 was constructed more often for the top-10 and -50 gene sets, but not for the top-100 gene set for both the Bian and Chen data. The PI peaks of these gene sets were closer to the common ancestral node of the teleost fish than the 412 gene set of the Bian data or the four sets of the Chen data (supplementary fig. S11, Supplementary Material online). The PI peak to the teleost common ancestral node of the top-10 gene set of the Chen data at the deep range of the teleost phylogeny (supplementary fig. S11*b*, Supplementary Material online) indicated the optimal power of this gene set for resolving the relationship regarding this node, whereas the peak of the top-10 gene set of the Bian data was in the shallower range than that of the Chen data (supplementary fig. S11*a*, Supplementary Material online). However, because the number of sites of this gene set was quite small (2,674), some of the constructed trees became unstable, matching none of Trees 1–3. Therefore, for the Bian data, even if a gene set with optimal low divergence could be created, because of the stochastic error caused by the small number of sites, it may not have the resolving power for Trees 1–3. In the case of the top-10 gene set of the Chen data, although Tree 2 was constructed for all the different outgroup species, the BPs supporting Tree 2 were not always high (≥45%). Therefore, in the Bian and Chen data, there were not enough genes with the optimal divergence to resolve the relationship of the three primary groups of teleost fish with high confidence.

Sequence divergence of the Hughes data was higher than those of the Bian data and Chen data (supplementary table S2, Supplementary Material online). In the PI approach, the peak of the power to resolve phylogenetic relationships was in the shallow range of the teleost fish (supplementary fig. S12, Supplementary Material online). The peak gradually moved to the deep range for the top-1000 to top-100 gene sets of low divergence whose TBLs are ∼90%–20% of the total gene set (supplementary table S2, Supplementary Material online). The peaks of the top-200 to top-100 gene sets appear to be in the deepest range of the teleost cluster.

For the gene sets of low divergence, Tree 1 was mostly constructed (table 6). However, with Holostei as the outgroup Tree 2 was constructed for the top1000- to top-400 gene sets. Note that in computer simulation, *N_C_* values were higher, using Holostei as the outgroup than the other outgroups for all the top-1000 to top-100 gene sets (supplementary table S12, Supplementary Material online).

**Table 6:** Phylogenetic Trees Constructed for Gene Sets with Small Sequence Divergence in the Hughes Data

Locus Set	No. Sites	Outgroup													
		All		Frog		Lungfish		Coelacanth		Polypteriformes		Acipenseriformes		Holostei	
		Tree	BP	Tree	BP	Tree	BP	Tree	BP	Tree	BP	Tree	BP	Tree	BP
All	166,583	1	100	1	100	1	100	1	100	1	100	1	100	1	73
Top-1000	147,894	1	93	1	100	1	100	1	100	1	100	1	100	2	50
Top-900	122,507	1	81	1	99	1	100	1	100	1	99	1	100	2	72
Top-800	106,454	1	69	1	98	1	100	1	100	1	98	1	97	2	77
Top-700	87,906	1^a^	68	1	95	1	100	1	96	1	99	1	93	2	66
Top-600	73,710	1^a^	43	1	84	1	98	1	89	1	91	1	84	2	78
Top-500	58,889	1	56	1	56	1	98	1	81	1	80	1	81	2	51
Top-400	47,018	3	72	3	54	1	74	1	51	3	56	1	41	2	82
Top-300	32,872	3	80	3	83	1	71	3	63	3	52	1	51	3	57
Top-200	20,936	3	85	3	67	1	49	3	79	1	58	1	47	3	85
Top-100	9,131	1	83	3	73	3	55	1	60	1	86	1	69	1	54

a Coelacanth and lungfish formed a cluster.

Tree 3 or Tree 1 was sometimes constructed for the top-400 to top-100 gene sets even when Holostei was used as the outgroup. This seems to be because the sampling error became large due to the small number of sites (<30% of the total gene set) (table 6). Correspondingly, the statistical support became generally low (BP ≤ 85%).

### Genes with Low Amino-Acid Frequency Bias

Heterogeneity of amino-acid frequencies among species is one of the factors that distorts tree topologies (Shen et al. 2016). The genes were also sorted by the average pairwise *χ*^2^ values of amino-acid frequencies between species and the top-10, -50, and -100 gene of small average *χ*^2^ values were obtained for the Bian data and Chen data (table 5). In the case of the Bian data, although most trees constructed for the top-10 gene set did not match any of Trees 1–3, the tree topologies constructed for the top-50 and -100 gene sets were similar to those for the top-50 and -100 gene sets of TBL. For the Chen data, although Tree 1 or Tree 3 was constructed for the top-50 gene set, Tree 2 was often constructed for the top-10 and -100 gene sets. Because the average *χ*^2^ value and TBL of genes were highly correlated (Pearson’s correlation coefficient = 0.76, *P *=* *2.4 × 10^−79^ for the Bian data and 0.81, *P *=* *4.8 × 10^−183^ for the Total set of the Chen data), it was difficult to separate the effect of low sequence divergence and homogeneity of amino-acid frequencies.

### GTRG Model and the Partitioned Approach

The GTRG substitution model and the partitioned approach were used for the phylogeny construction of concatenated sequences of the Bian and Chen data, in addition to the JTTFG model. For the Bian data, the log-likelihood value (*L*) using the partitioned approach with AICc was the highest, except in one case in which *L* for the GTRG model was the highest (supplementary table S13, Supplementary Material online), whereas for the Chen data, *L* for the GTRG model was the highest, except for several cases in which *L* was the highest using partitioning according to AICc (supplementary table S14, Supplementary Material online). However, by penalizing the *L* value with the number of parameters estimated (*k*) in AICc (= –2 *L *+* *2*k* + 2*k*(*k *+* *1)/(*n–k* – 1)), where *n* is the number of sites in the alignment, the GTRG had the best fit in the majority of the cases for both sets of data, having the smallest AICc value. However, the partitioned approach with BIC had the best fit in a small number of cases (supplementary tables S13 and S14, Supplementary Material online). Therefore, whether the GTRG or the partitioned approach has a better fit depended on the data sets used. However, the results for the GTRG and the partitioned approach tree topologies did not differ, except for the cases in which statistical support was low (BP ≤ 55%) (supplementary table S7, Supplementary Material online). Thus, for the Bian and Chen data, the effect of using the GTRG or the partitioned approach for tree construction was not strong, in contrast to the use of different species and genes, wherein tree topologies changed even in cases with high statistical support (table 5 and supplementary table S7, Supplementary Material online).

### Effects of Species and Genes on Tree Topologies

To improve the accuracy of the constructed phylogeny, choice of species and genes and models used for the analysis were tested. However, there is no concrete measure that can quantitatively evaluate the power of the data by taking into accounts the effect of species and genes, and choose the optimal data for resolving a phylogenetic relationship (López-Giráldez et al. 2013).

Exclusion of fast-evolving species or outgroups can mitigate the effect of long branch attraction (Philippe, Delsuc, et al. 2005), but it was only done to see the change in tree topology with their progressive removal (e.g., Brinkmann et al. 2005; Pisani et al. 2015). Importance of rich taxon sampling (e.g., Dunn et al. 2008; Philippe et al. 2009; Whelan et al. 2015) was indicated by the theoretical study (Townsend and López-Giráldez 2010) and computer simulations (Hillis 1998; Heath et al. 2008). However, the efficiency of the conditions for species addition or exclusion for tree construction is not known (Hillis et al. 2003; Rosenberg and Kumar 2003; Brinkmann et al. 2005).

Various properties of genes have been used to choose them for tree construction. Genes with high divergence (e.g., Brinkmann et al. 2005; Betancur-R et al. 2014; Whelan et al. 2015) were excluded to reduce the effect of multiple substitutions, which may obscure the phylogenetic signals. There are other gene properties, such as strong phylogenetic signals (e.g., high bootstrap values, Salichos and Rokas 2013, and high information content, Meusemann et al. 2010) and composition bias (Collins et al. 2005). However, the effect of different gene properties varies with the data used and the depth of the node to be resolved (López-Giráldez et al. 2013; Shen et al. 2016). Shen et al. (2016) investigated the relationship between measures of tree topology resolution and various gene properties, such as variability (e.g., pairwise sequence identity, number of parsimony-informative sites, number of variable sites), composition bias, codon usage, gene interaction, protein abundance, and internal branch lengths in yeast and mammalian data and found that only gene properties that consistently contributed to tree topology resolution for both data were sequence length and compositional variability among species.

Therefore, commonly used criteria or measures for choosing species and genes may not necessarily provide the data with sufficient resolving power for a particular phylogenetic relationship. Even among the studies whose data were used in this study, Bian et al. (2016) improved taxon sampling by adding tarpon in Elopomorpha, and knifefish and butterflyfish in Osteoglossomorpha; Chen et al. (2015) filtered genes by high information content, low evolutionary rate, high resolution of branching patterns by bootstrap value, and appearance of the teleost fish clade in gene trees.

However, this study indicated that the use of the species/outgroup with low divergence and slowly evolving genes increased the resolving power of phylogenetic relationship of the primary groups of the teleost fish and that there are not sufficiently large numbers of genes with optimal sequence divergence to resolve this node with statistical support for the Bian, Chen, and Hughes data. Therefore, rather than excluding fast-evolving genes with a hope to increase the resolving power for the entire phylogeny, choosing genes with optimal divergence with respect to a node of interest specifically (Townsend 2007; López-Giráldez et al. 2013) could be a promising way to increase the resolving power of the data.

## Supplementary Material

Supplementary data are available at *Genome Biology and Evolution* online.

## Supplementary Material

evab052_Supplementary_Data
