# Corrigendum to: Resolving the Early Divergence Pattern of Teleost Fish Using Genome-Scale Data

**DOI:** 10.1093/gbe/evab118

**Published:** 2021-06-19

**Authors:** Naoko Takezaki

In the originally published version of this manuscript, there was an error in the journal name for the Johnson GD reference. The full correct reference is: Johnson GD, et al. 2012. A ‘living fossil’ eel (Anguilliformes: protanguillidae, fam. nov.) from an undersea cave in Palau. Proc R Soc B. 279:934–943.

This error has been corrected online.

## Notes

*Genome Biology and Evolution*, Volume 13, Issue 5, May 2021, evab052, https://doi.org/10.1093/gbe/evab052

