# Supplementary material for: Resolving the Early Divergence Pattern of Teleost Fish Using Genome-Scale Data
Source: Genome Biol Evol. 2021 Mar 19;13(5):evab052. doi: 10.1093/gbe/evab052 (PMC8103497; doi:10.1093/gbe/evab052)
Supplement: evab052_Supplementary_Data [file evab052_supplementary_data.zip › figureSup.pdf]

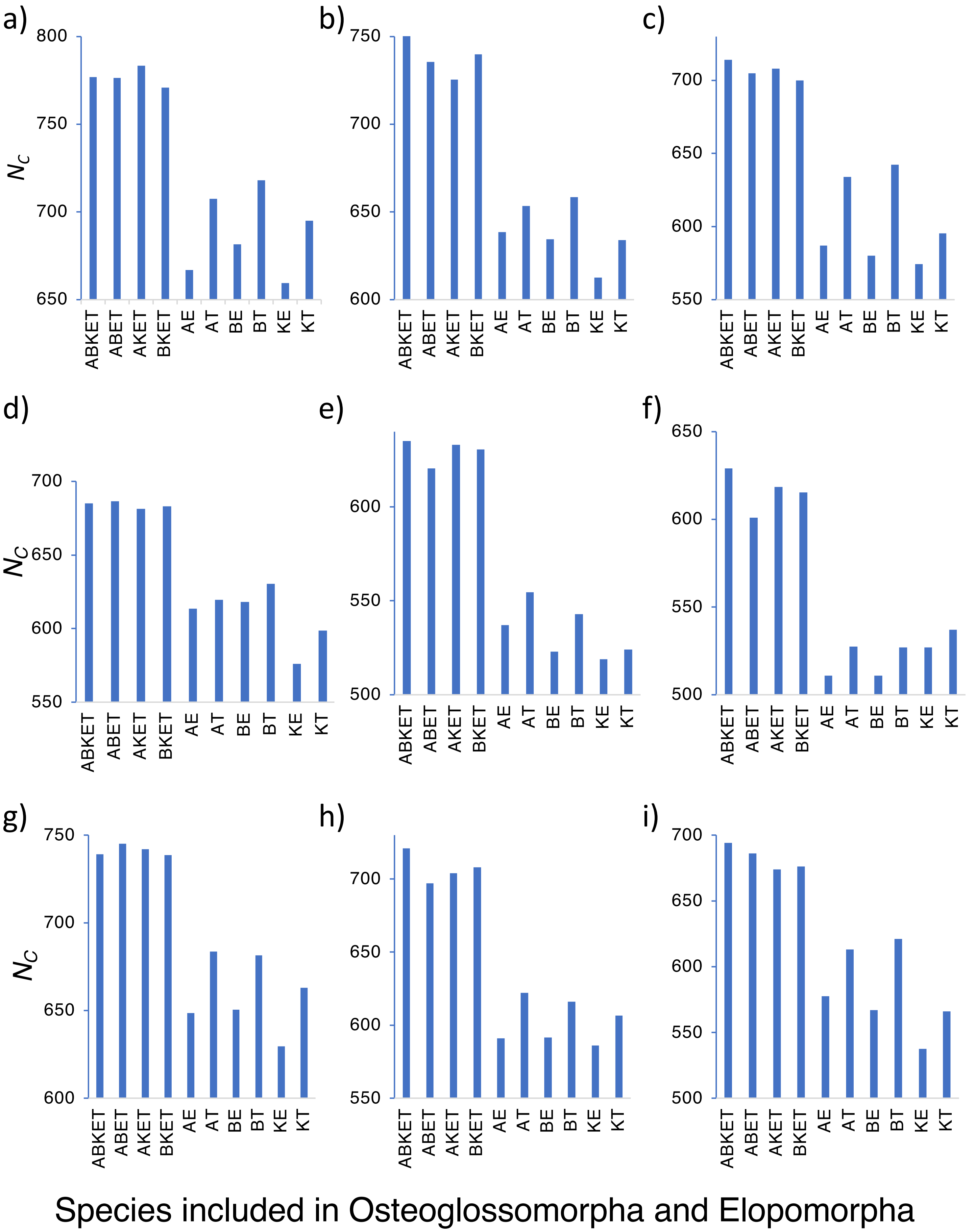

Figure S1. The numbers of replications in which the correct tree topologies were obtained (*Nc*) in the cases in which multiple species were included in Osteoglossomorpha and Elopomorpha and in the cases in which one species was included in Osteoglossomorpha and Elopomorpha for the Bian data. Outgroups used were coelacanth and gar in a) – c), coelacanth in d) - f) , and gar in g) – i). as the outgroup. The model tree was Tree 1 in a), d), g), Tree 2 in b), e), h), and Tree 3 in c), f), i).

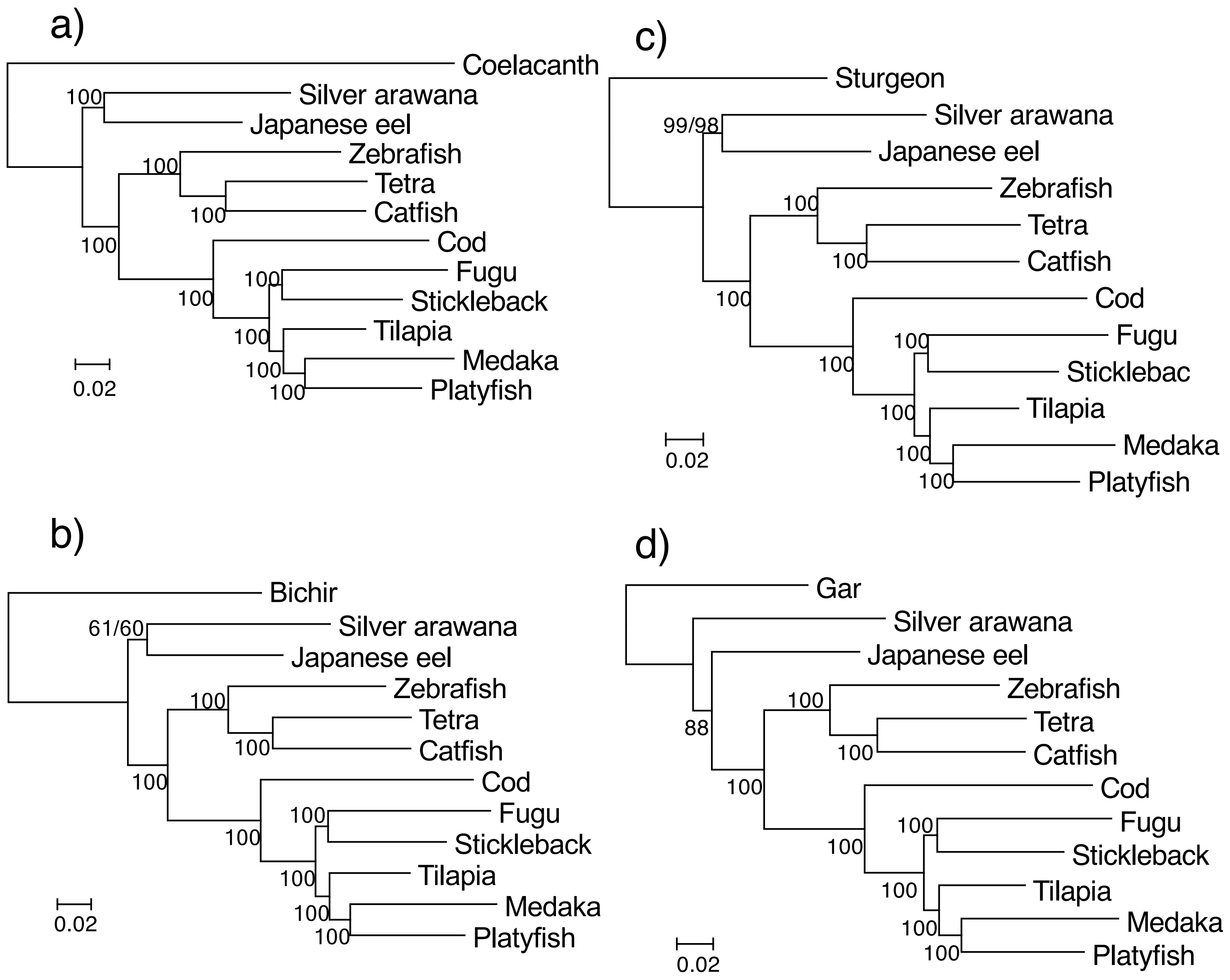

Figure S2. The phylogenetic trees constructed for concatenated sequences of the Total set from Chen data. JTTFG was used and 500 bootstrap replications were done. Coelacanth, bichir, sturgeon and gar were used as outgroups in a), coelacanth in b), bichir in c), sturgeon in d) and gar in e). The BP for GTRG is shown after the slash if it is different from that for JTTFG.

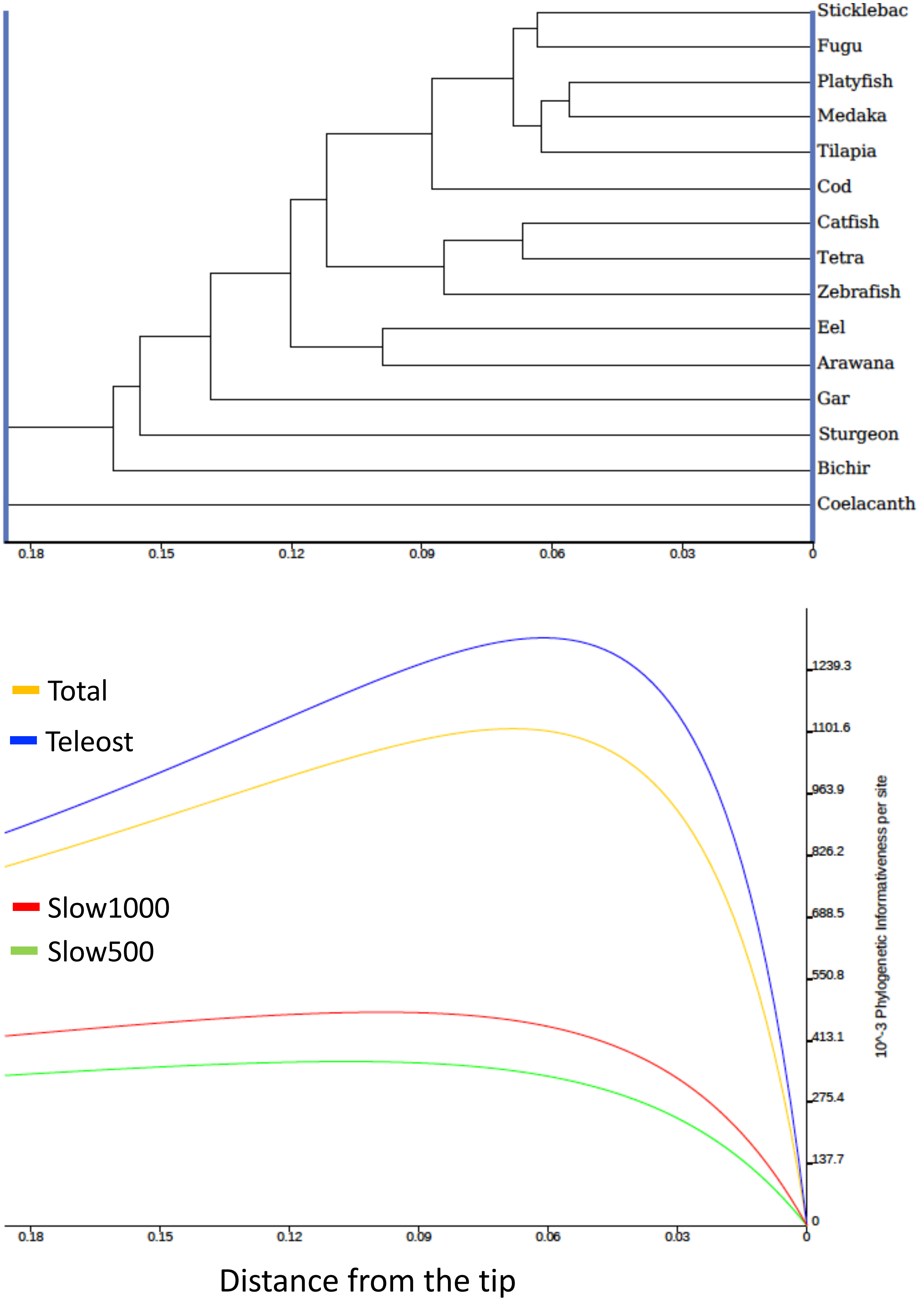

Figure S3. Profile of phylogenetic informativeness for the four sets of the Chen data. Per-site PI values are shown.

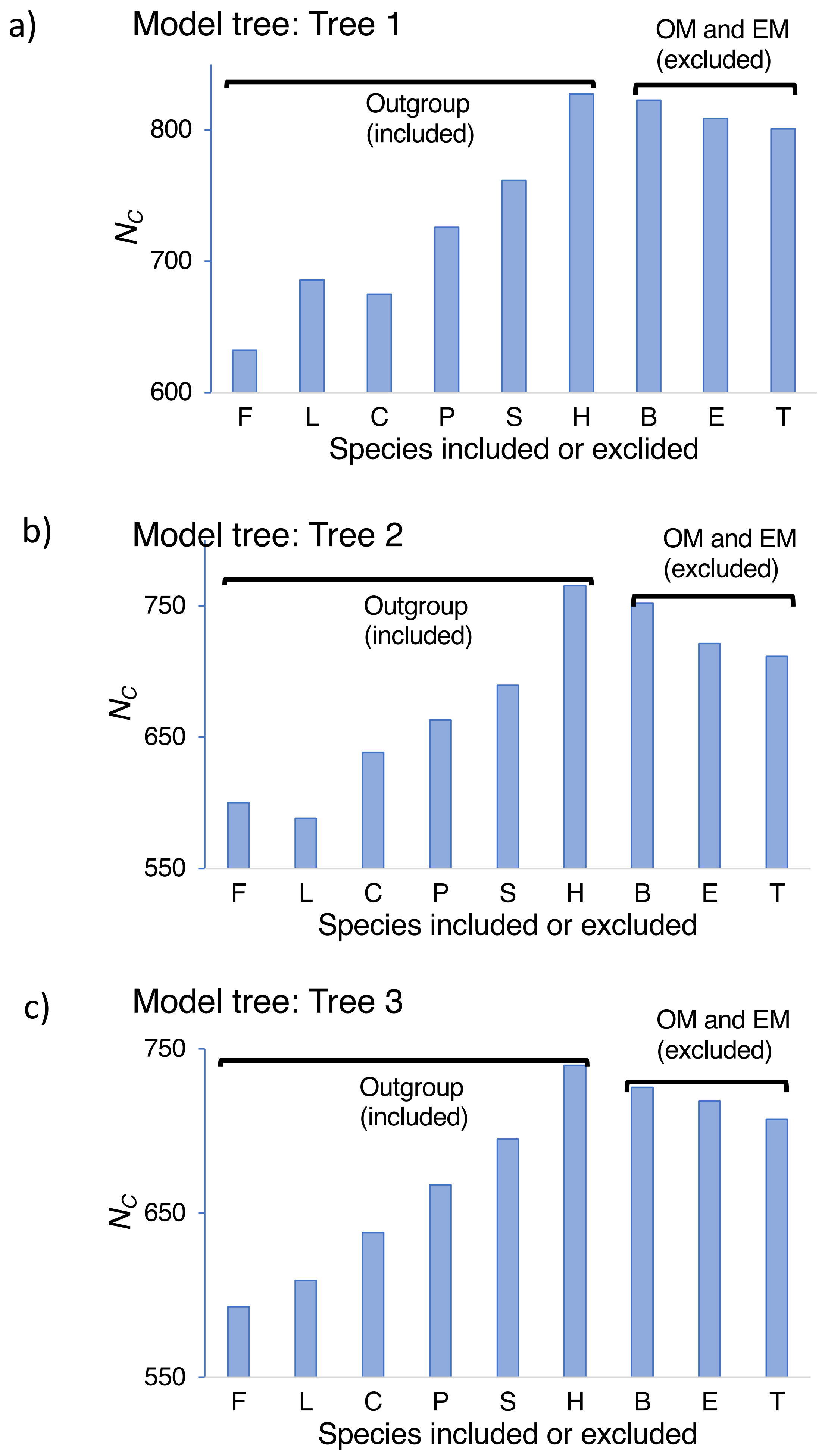

Figure S4. The number of replications in which correct tree topologies are obtained (N_C) in computer simulation for the Hughes data. Model tree used was Tree 1 in (a), Tree 2 in (b) and Tree 3 in (c). Species in the four groups, outgroup, EM, and OM were changed. Outgroup: F, frog, L, lungfish, C, coelacanth, P, Polypteryformes, S, Acipenseriformes, H, Holostei. EM: E, eels, T , tarpon. OM: B, butterflyfish.

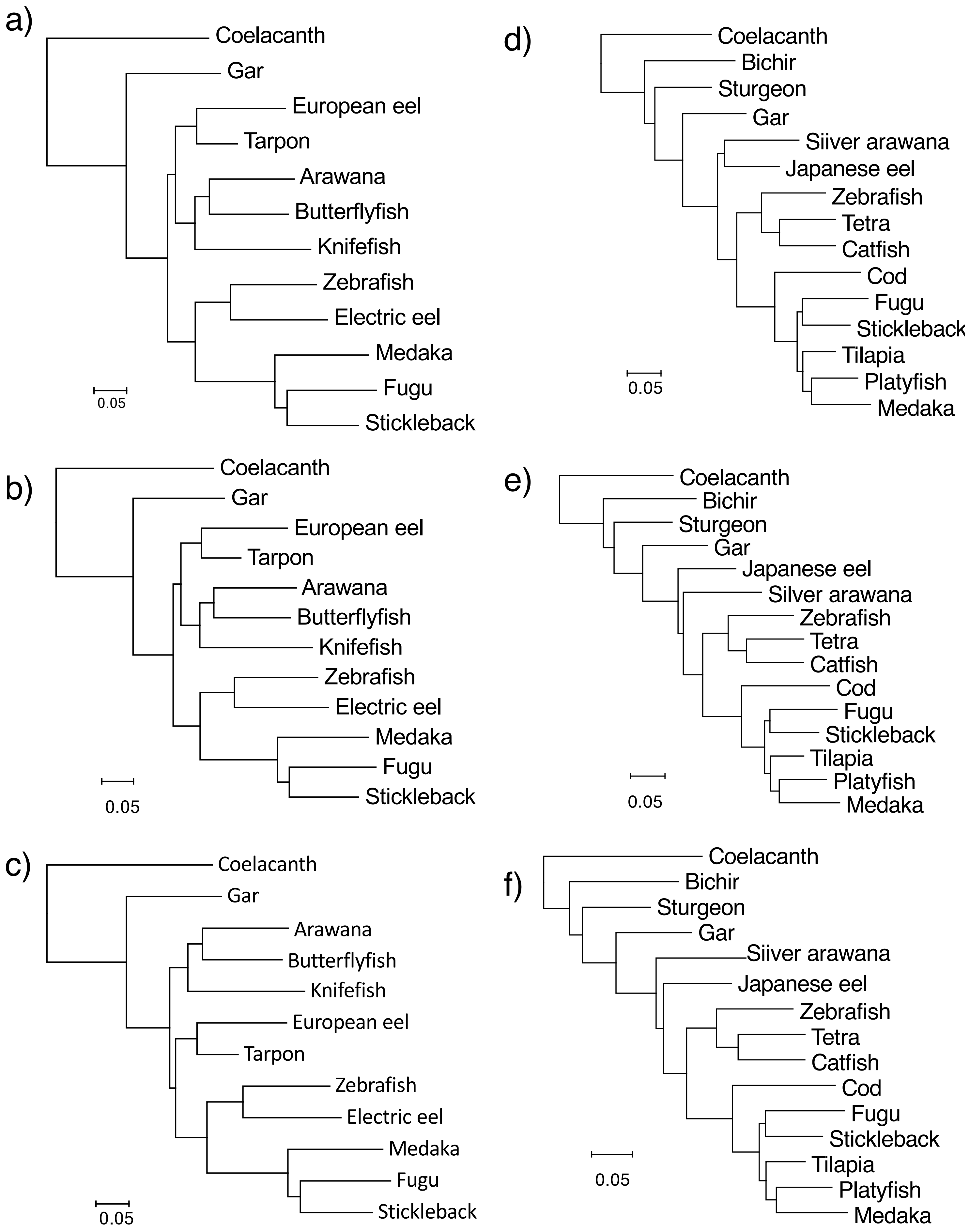

Figure S5. Model trees used for computer simulation. The branch lengths were estimated by assuming the tree topologies shown above for the concatenated sequences of 412 genes of the Bian data and 772 genes of the Total set of Chen data. with JTTFG. a), d) Tree 1. b), e) Tree 2. c), f) Tree 3.

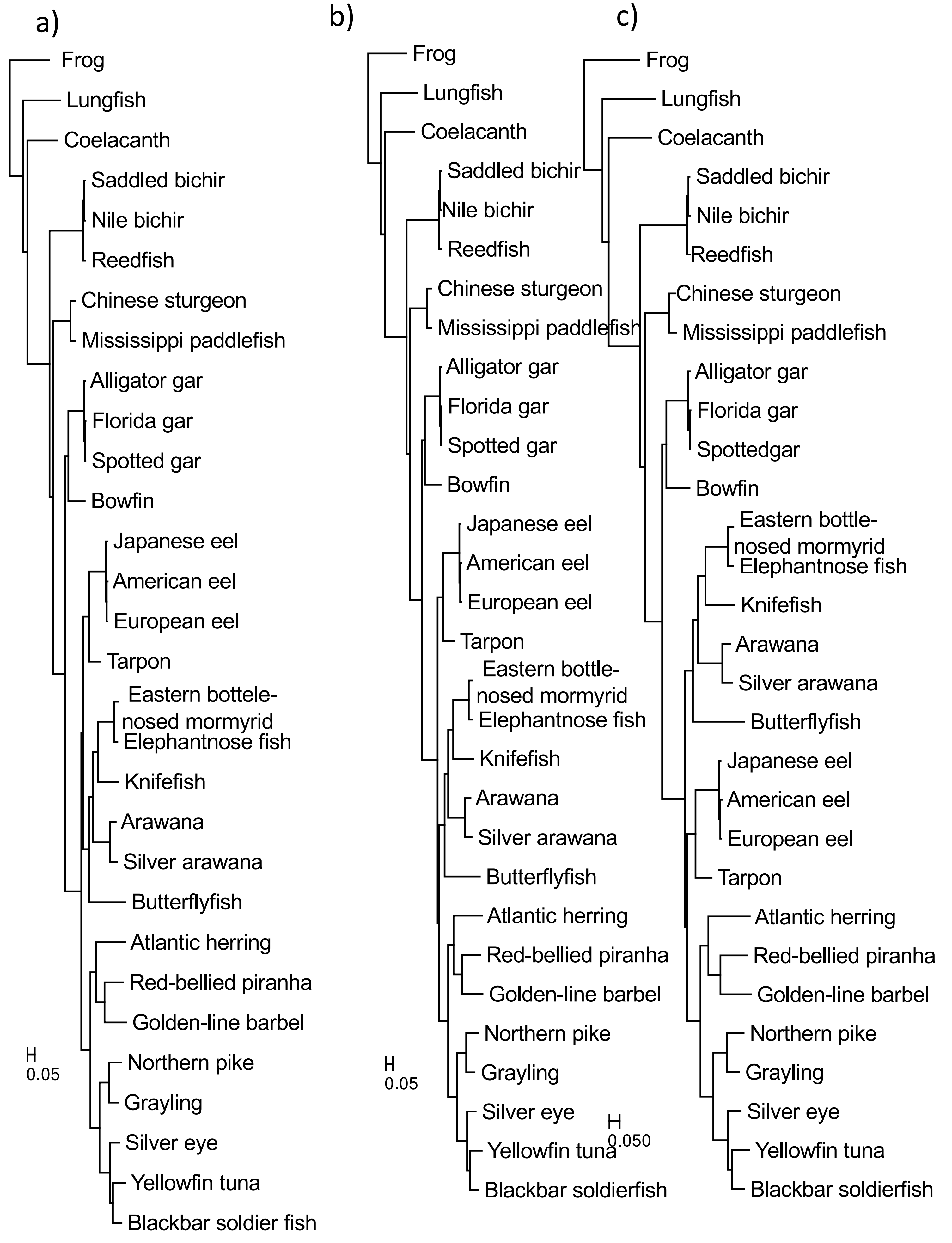

Figure S6. Model trees used for computer simulation. The branch lengths were estimated by assuming the tree topologies shown above for the concatenated sequences of 1,062 genes of the Hughes data with JTTFG. a) Tree 1. b) Tree 2. c) Tree 3.

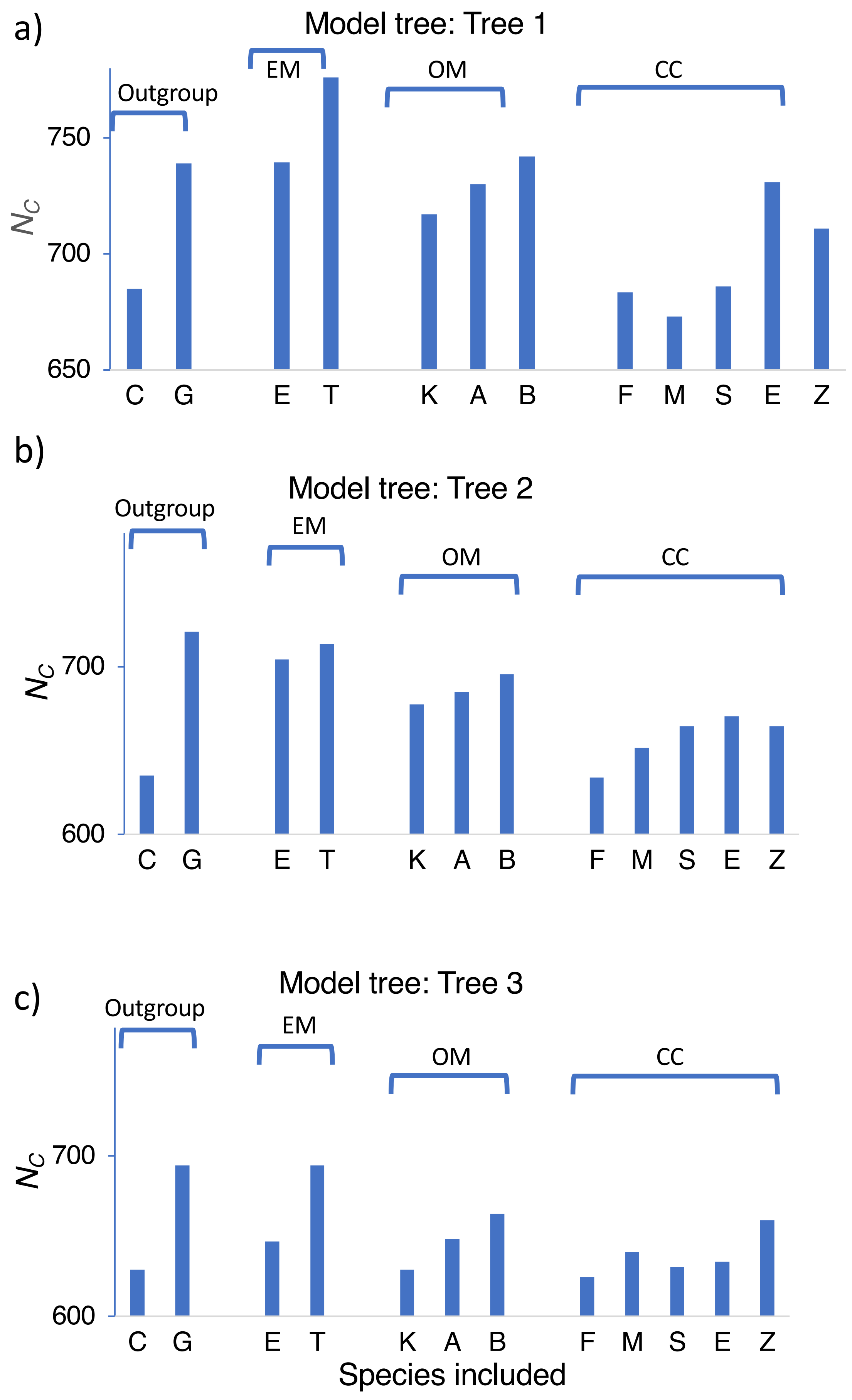

Figure S7. The number of replications in which correct tree topologies are obtained (N_C) in computer simulation for the Bian data. Model tree used was Tree 1 in (a), Tree 2 in (b) and Tree 3 in (c). Species in the four groups, outgroup, EM, OM, and CC, were changed. Outgroup: C, coelacanth, G, gar. EM: E, European eel, T , tarpon. CC: Z, zebrafish, E, Electric eel, S, stickleback, F, fugu, M, medaka.

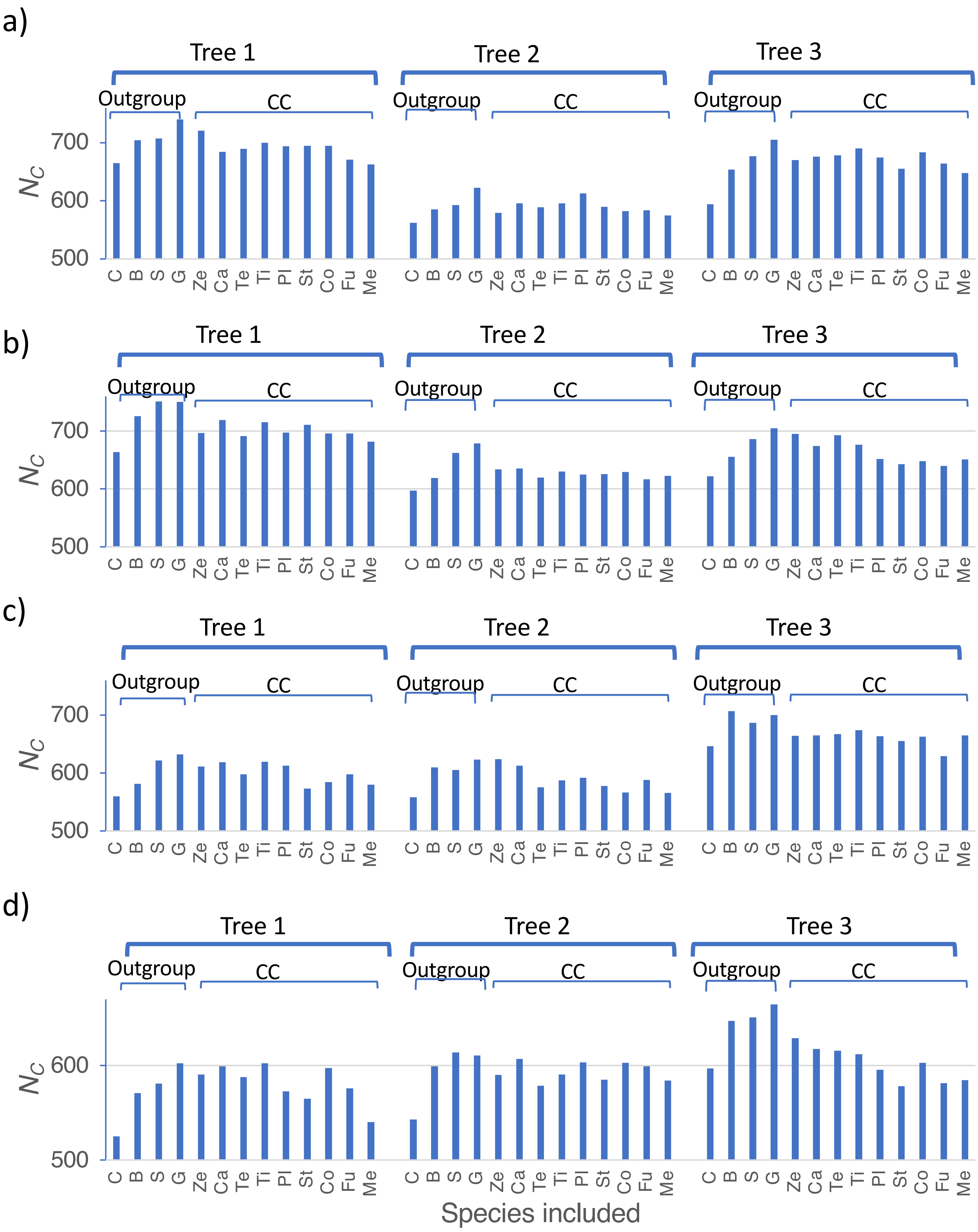

Figure S8. The number of replications in which correct tree topologies are obtained (N_C) in computer simulation for the Chen data. (a). Total set. (b) Teleost set. (c) Slow1000 set. (d) Slow500 set. Species in outgroup and CC were changed. Outgroup: C, coelacanth, B, bichir, S, sturgeon, and G, gar. CC: Zeb, zebrafish, Cat, catfish, Tet, tetra, Til, tilapia, Sti, stickleback, Pla, platyfish, Cod, cod, Fug, fugu, and Med, medaka. When species of one group was changed, all the species in the other groups were included. The result of the cases in which Tree 1 was used as the model tree.

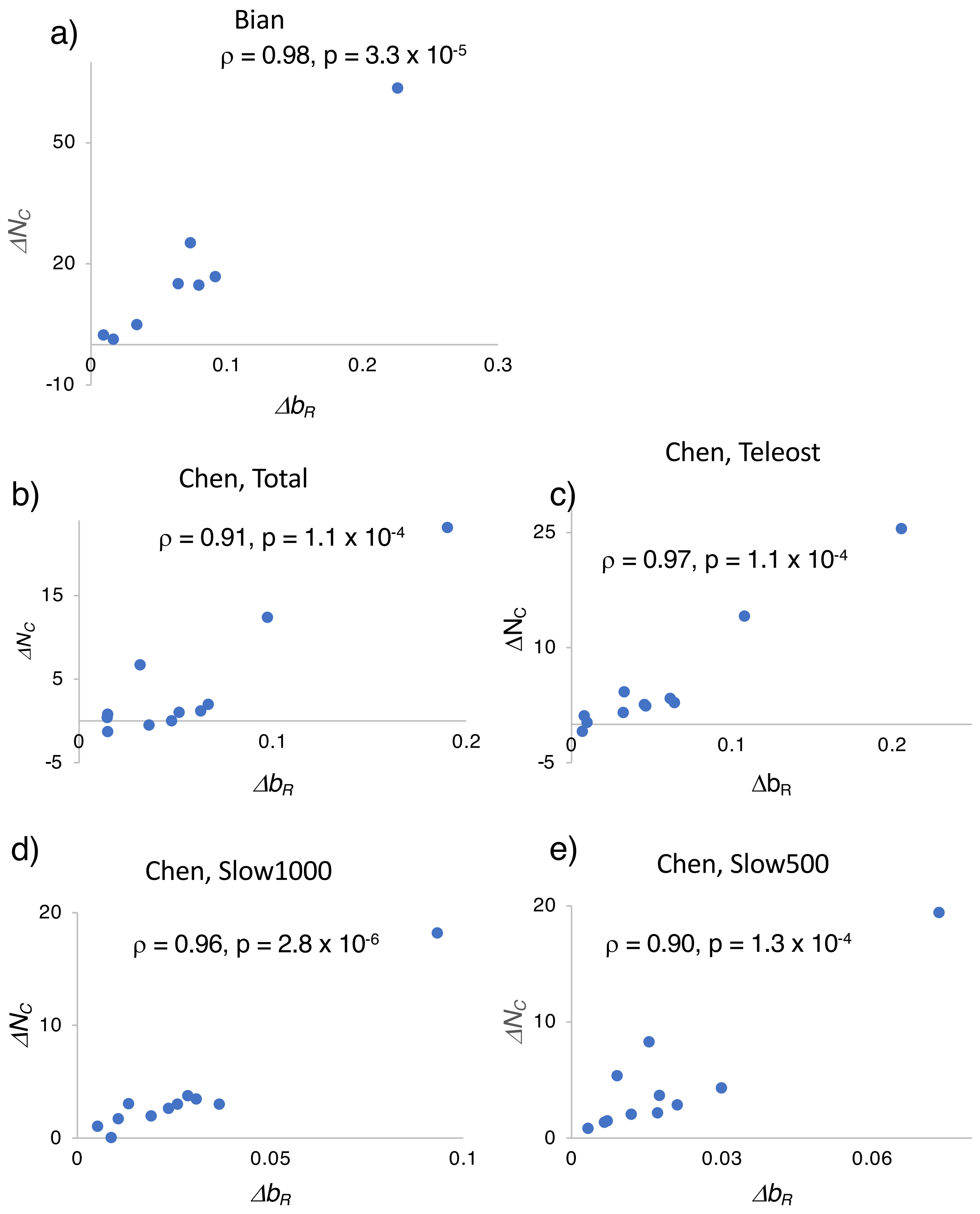

Figure S9. The relationship of difference in the number of replications in which correct tree topologies are obtained (ΔN_C) and the difference of branch lengths from the common ancestral node of teleost fish (Δb_R) in computer simulation. a): Bian data. b)- e): Chen data. ΔN_C is the average of the result of model tree 1 – 3 and the cases corresponding to the cases in which Δb_R were calculated. The Δb_Rs shown are those of gar from coelacanth, arawana and knifefish from butterflyfish, and electric eel, stickleback, medaka, and fugu from zebrafish in the Bian data, and sturgeon, bichir, coelacanth from gar, and catfish, tetra, tilapia, stickleback, platyfish, cod, fugu, and medaka from zebrafish for the Chen data. See for the values in Table S16. Pearson's correlation coefficient is shown in the graph.

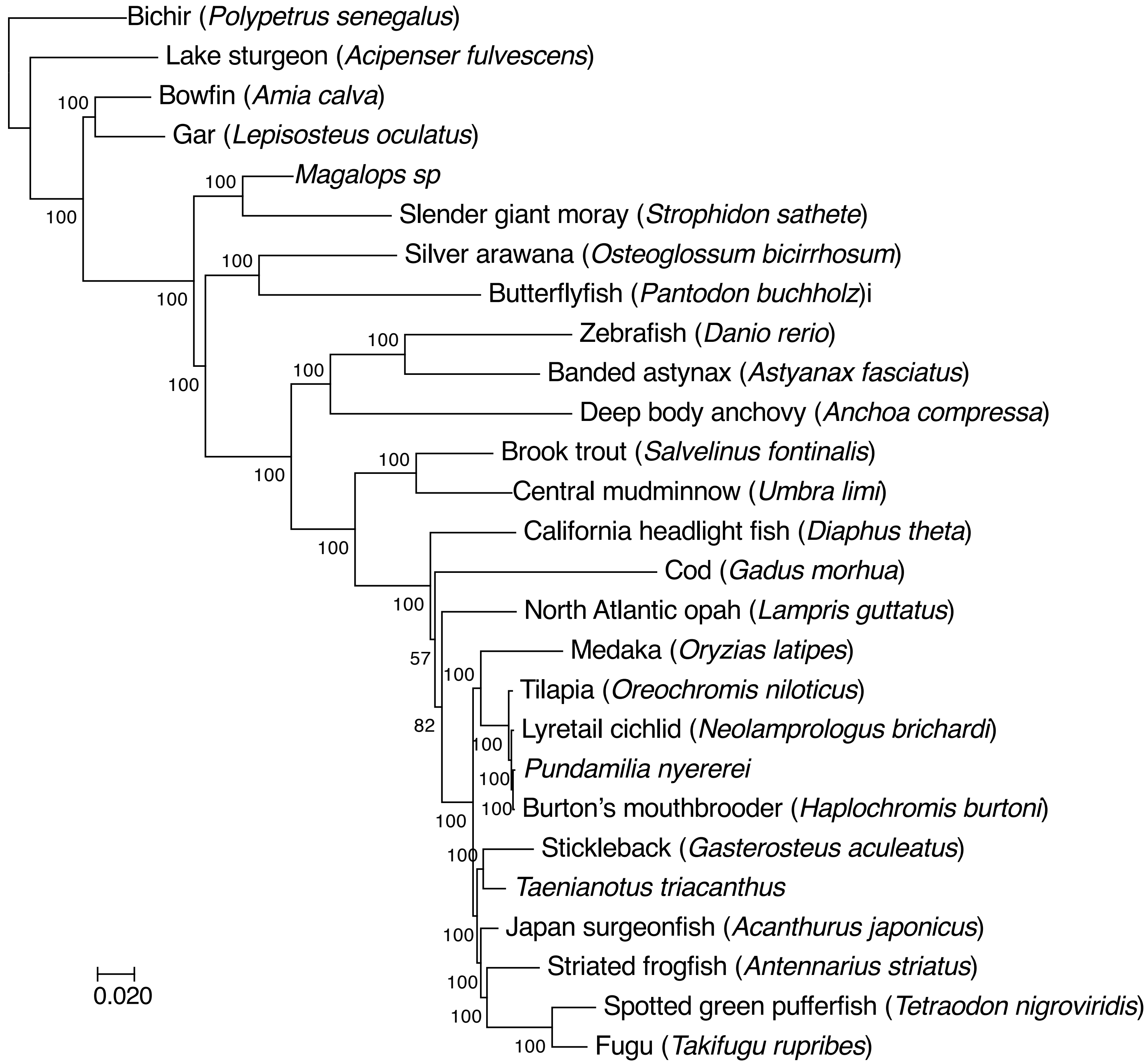

Figure S10. Phylogenetic tree constructed for concatenated sequence of the UCE data. The tree was constucted by the maximum likelihood method with GTRG model using RAXML. 500 bootstrap relications were carried out. The UCE data is from Faircloth et al. (2013).

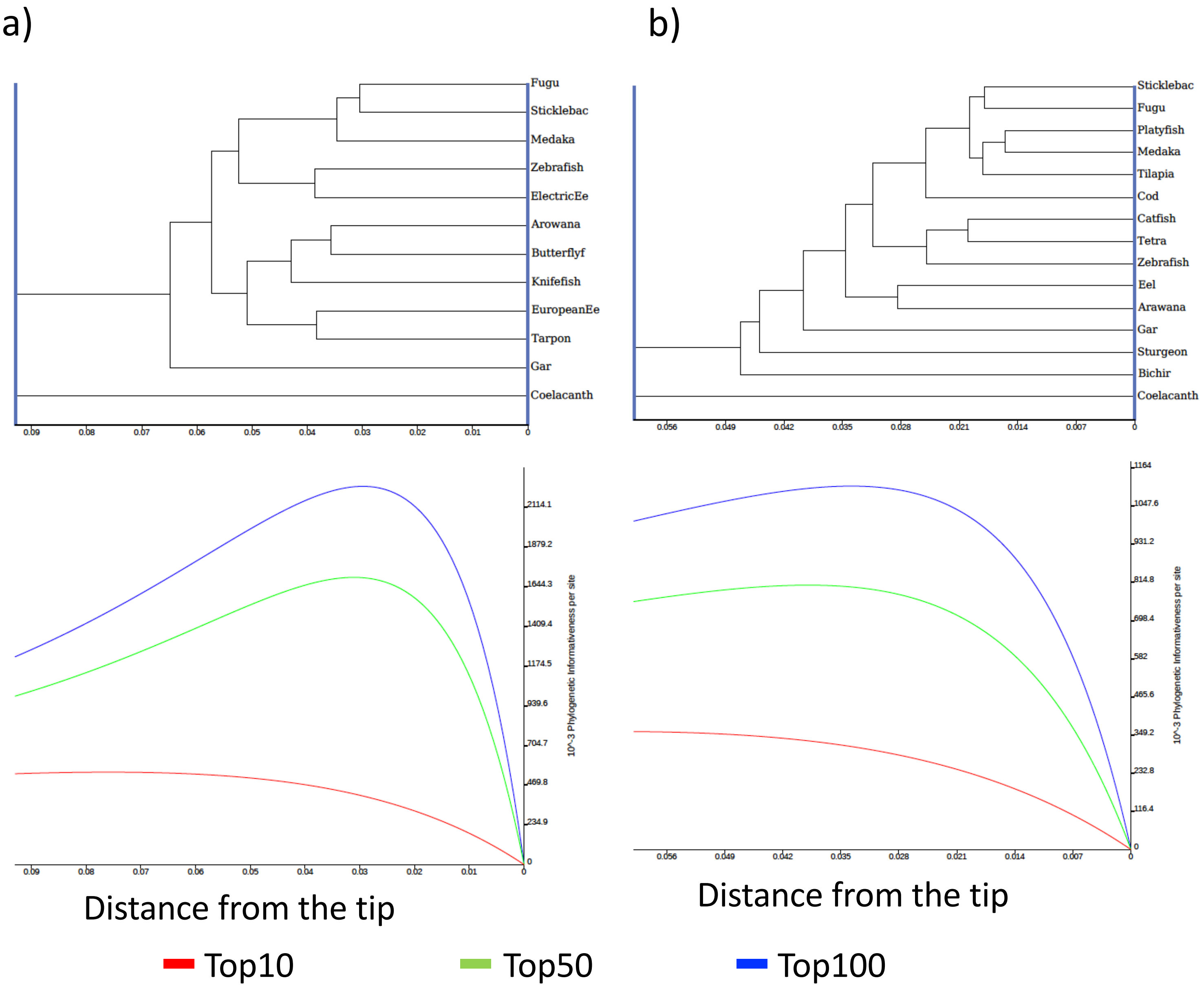

Figure S11. Profiles of phylogenetic informativeness for top-10, -50, and, -100 gene sets of small divergence for the Bian and Chen data. a) Bian data. b) Chen data. Per-site PI values are shown. The site rates were justified by multiplying them the ratio of the branch length estimated for the ultrametric tree for the set to that for the Top-100 set. Species names are abbreviated as follows. Sticklebac, stickleback; ElectricEe, electric eel; Butterflyf, butterflyfish; EuropeanEe, European eel.

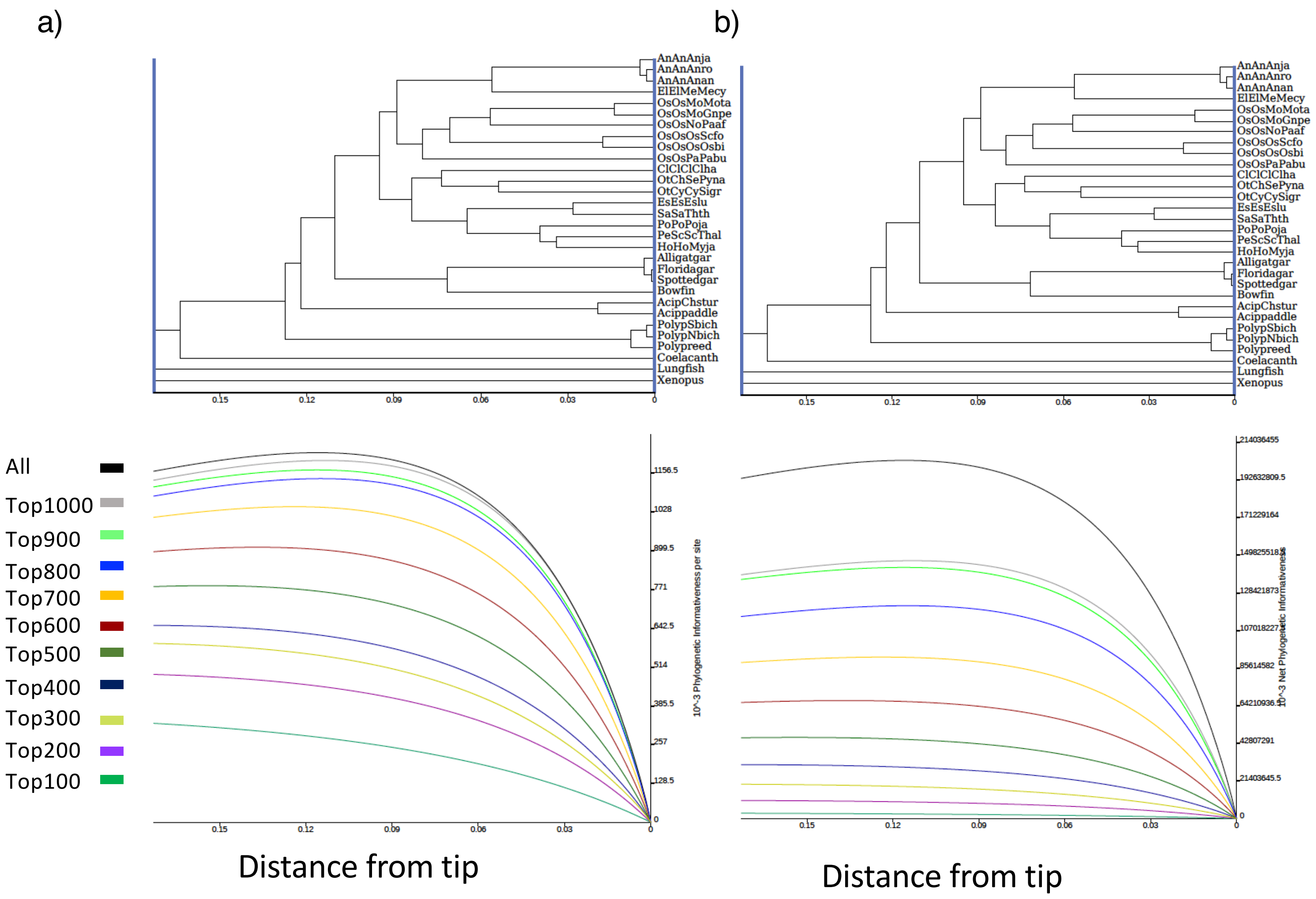

Figure S12. Profiles of the phylogenetic informativeness for the top-1000 to top-100 gene sets of low divergence for the Hughes data. a) Per-site PI values. b) PI values. Species names are abbreviated as follows: AnAnAnja, Japanese eel; AnAnAnro, American eel; European eel; ElElMeMecy, tarpon; OsOsMoMota, eastern bottle-nosed mormyrid; OsOsMoGnpe, elephantnose fish; OsOsNoPaaf, knifefish; OsOsOsScfo, arawana; OsOsOsOsbi, silver arawana; OsOsPaPabu, butterflyfish; ClClClClha, Atlantic herring; OtChSePyna, red-bellied piranha; OtCyCySigr, golden-line barbel; EsEsEslu, northern pike; SaSaThth, grayling; PoPoPoja, silver eye; PeScScThal, yellowfin tuna; HoHoMyja, blackbar soldierfish; Alligatgar, alligator gar; Floridagar, Florida gar; Spottedgar, spotted gar; AcipChstur, Chinese sturgeon; Acippaddle, Mississippi paddlefish; PolypSbich, saddled bichir; PolypNbich, Nile bichir; Polypreed, reedfish.

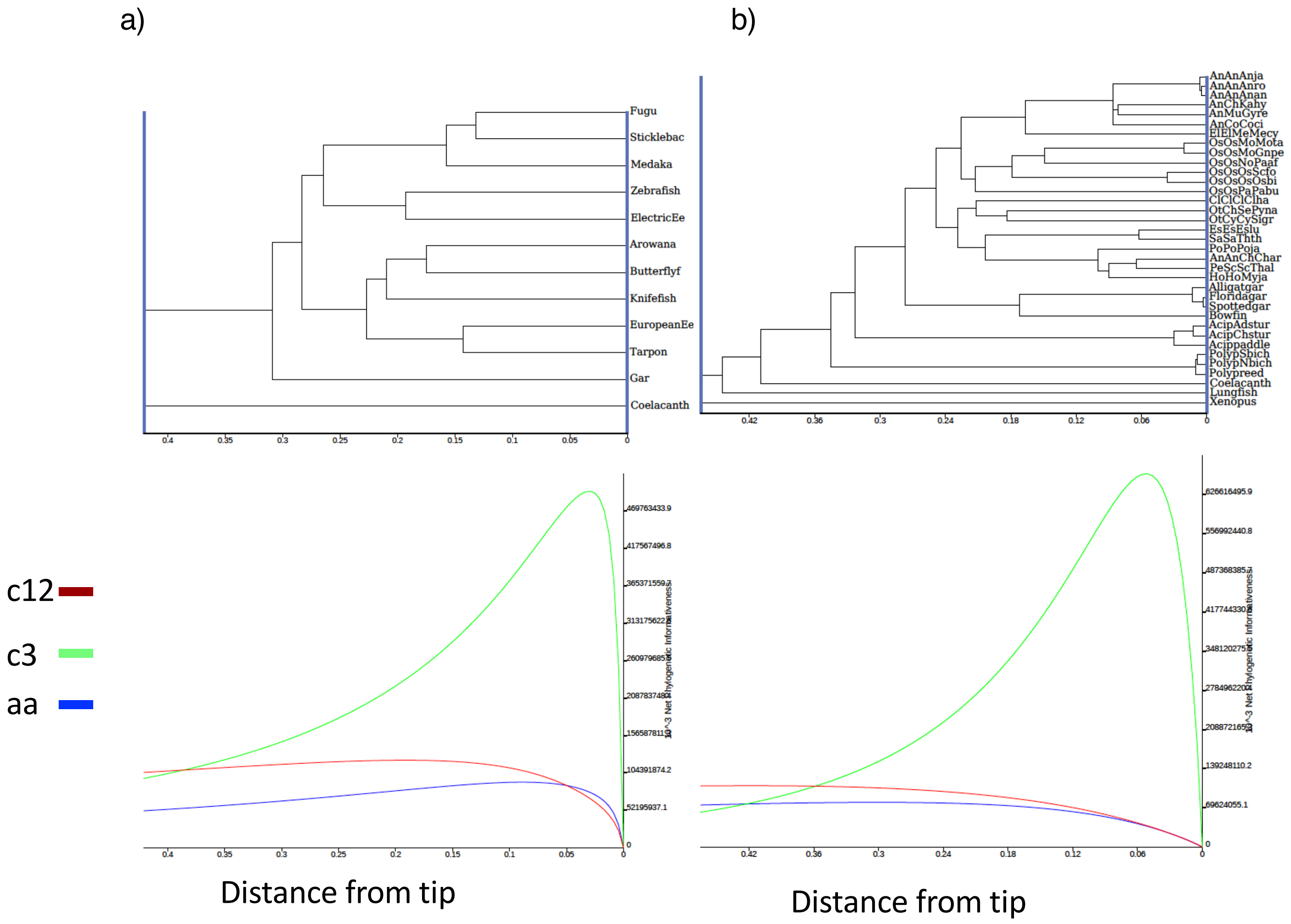

Figure S13. Profiles of the phylogenetic informativeness for the first and second codon positions, the third codon positions and amino acid sequence for the Bian and the Hughes data. a) Bian data. b) Hughes data. c12: first and second codon positions. c3: third codon positions. aa: amino acid sequence. Species names are abbreviated as follows. For the Bian data: Sticklebac, stickleback; ElectricEe, electric eel; Butterflyf, butterflyfish; EuropeanEe, European eel. For the Hughes data: AnAnAnja, Japanese eel; AnAnAnro, American eel; European eel; ElElMeMecy, tarpon; OsOsMoMota, eastern bottle-nosed mormyrid; OsOsMoGnpe, elephantnose fish; OsOsNoPaaf, knifefish; OsOsOsScfo, arawana; OsOsOsOsbi, silver arawana; OsOsPaPabu, butterflyfish; ClClClClha, Atlantic herring; OtChSePyna, red-bellied piranha; OtCyCySigr, golden-line barbel; EsEsEslu, northern pike; SaSaThth, grayling; PoPoPoja, silver eye; PeScScThal, yellowfin tuna; HoHoMyja, blackbar soldierfish; Alligatgar, alligator gar; Floridagar, Florida gar; Spottedgar, spotted gar; AcipChstur, Chinese sturgeon; Acippaddle, Mississippi paddlefish; PolypSbich, saddled bichir; PolypNbich, Nile bichir; Polypreed, reedfish.

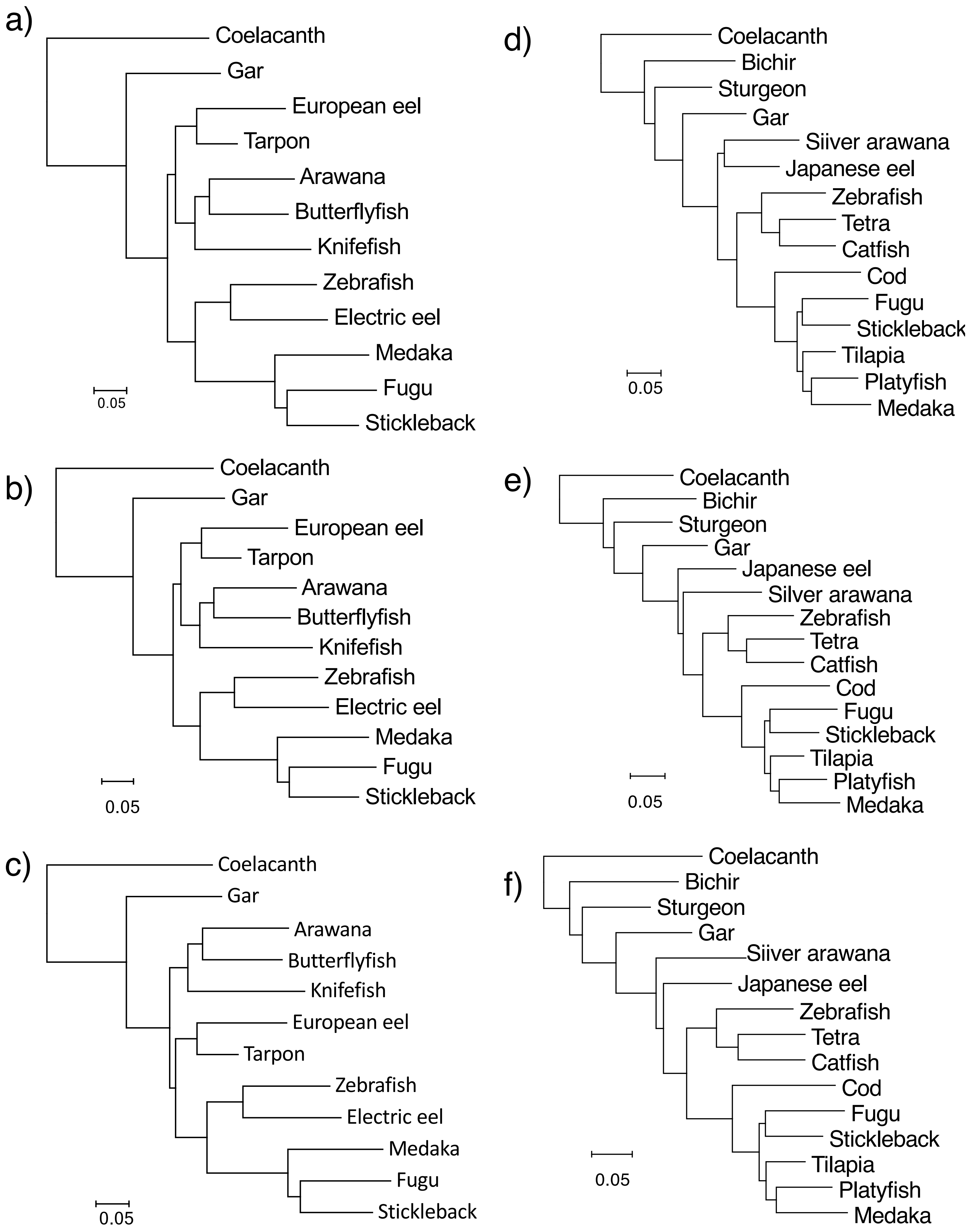

Figure S14. Model trees used for computer simulation. The branch lengths were estimated by assuming the tree topologies shown above for the concatenated sequences of 412 genes of the Bian data and 772 genes of the Total set of Chen data. with JTTFG. a), d) Tree 1. b), e) Tree 2. c), f) Tree 3.

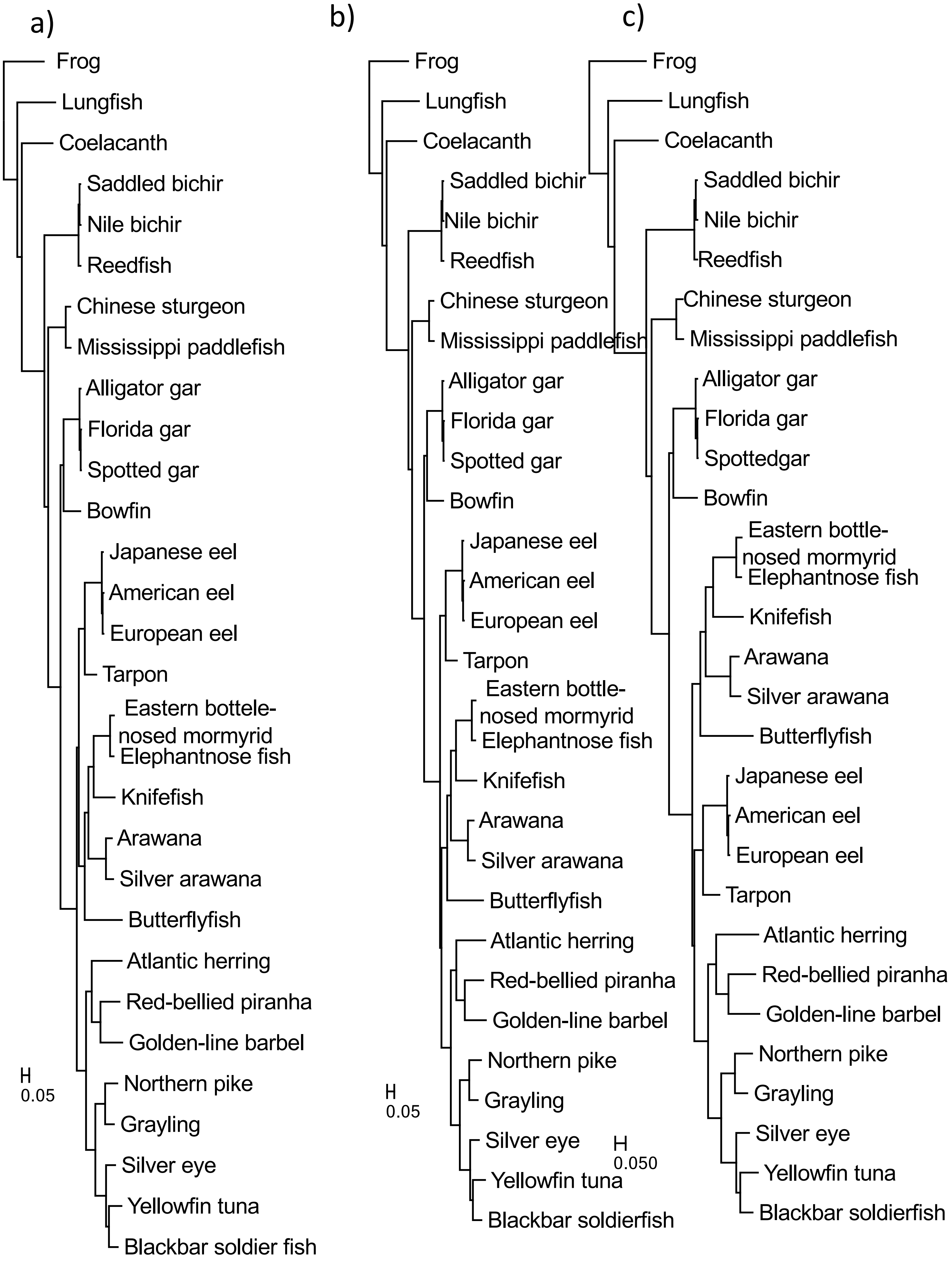

Figure S15. Model trees used for computer simulation. The branch lengths were estimated by assuming the tree topologies shown above for the concatenated sequences of 1,062 genes of the Hughes data with JTTFG. a) Tree 1. b) Tree 2. c) Tree 3.
